# Novel Zinc(II) Complexes of Heterocyclic Ligands as Antimicrobial Agents: Synthesis, Characterisation, and Antimicrobial Studies

**DOI:** 10.1155/2014/276598

**Published:** 2014-02-23

**Authors:** Ramesh S. Yamgar, Y. Nivid, Satish Nalawade, Mustapha Mandewale, R. G. Atram, Sudhir S. Sawant

**Affiliations:** ^1^Department of Chemistry, Chikitsak Samuha's Patkar-Varde College of Arts, Science and Commerce, Goregaon (W), Mumbai 400 062, India; ^2^P. G. Department of Chemistry, Government of Maharashtra, Ismail Yusuf Arts, Science and Commerce College, Jogeshwari (East), Mumbai 400 060, India

## Abstract

The synthesis and antimicrobial activity of novel Zn(II) metal complexes derived from three novel heterocyclic Schiff base ligands 8-[(*Z*)-{[3-(N-methylamino)propyl]imino}methyl]-7-hydroxy-4-methyl-2*H*-chromen-2-one, 2-[(*E*)-{[4-(1*H*-1,2,4-triazol-1-ylmethyl)phenyl]imino}methyl]phenol, and (4*S*)-4-{4-[(*E*)-(2-hydroxybenzylidene)amino]benzyl}-1,3-oxazolidin-2-one have been described. These Schiff base ligands and metal complexes are characterised by spectroscopic techniques. According to these data, we propose an octahedral geometry to all the metal complexes. Antimicrobial activity of the Schiff base ligand and its metal complexes was studied against Gram negative bacteria: *E. coli* and *Pseudomonas fluorescens*, Gram positive bacteria: *Staphylococcus aureus,* and also against fungi, that is, *C. albicans* and *A. niger*. Some of the metal complexes show significant antifungal activity (MIC < 0.2 **μ**g/mL). The “*in vitro*” data has identified [Zn(NMAPIMHMC)_2_]*·*2H_2_O, [Zn(TMPIMP)_2_]*·*2H_2_O, and [Zn(HBABO)_2_]*·*2H_2_O as potential therapeutic antifungal agents against *C. albicans* and *A. niger*.

## 1. Introduction

The treatment of infectious diseases still remains an important and challenging problem because of various factors like emerging infectious diseases and the increasing number of multidrug resistant microbial pathogens. In recent years, bacterial resistance to antibiotics has been a matter of great concern. Antibiotic resistance is the ability of bacteria or other microbes to resist the effects of an antibiotic. Antibiotic resistance occurs when bacteria change in some way that reduces or eliminates the effectiveness of drugs designed to cure or prevent infections. The bacteria survive and continue to multiply causing more harm [1]. Bacteria can do this through several mechanisms like mutation of their genome or by accepting antimicrobial resistant genes from other bacteria. This usually occurs through one of several biochemical mechanisms like mutation, destruction, or inactivation and efflux.

Due to increasing resistance of these bacterial strains, effective antibacterial medicines like Vancomycin, Ciprofloxacin, Methicillin, and so forth become less effective in treatment of diseases caused by such infections. Over the past several decades, the incidence of resistant Gram positive organisms has risen in the world. Methicillin, a resistant *Staphylococcus aureus* (MRSA), is of special concern in regard to treatment because it is usually multidrug resistant. In addition to most beta-lactams, MRSA is also commonly resistant to clindamycin, erythromycin, fluoroquinolones, aminoglycosides, cotrimoxazole, and rifampicin. These situations have revealed a substantial medical need for discovery of new classes of compounds endowed with antimicrobial activities.

To overcome such challenges in treating patients with infections of such antibacterial resistant strains, new antimicrobial agents, that is, new medicines, need to be researched and continuous efforts are necessary to explore small molecular structures as new medicines. A lower molecular weight cutoff of 500 Daltons (as part of Lipinski's “rule of five”) [2] has been recommended for small molecule drug development candidates based on the observation of clinical attrition rates. A small molecule is a low molecular weight (<900 Daltons) compound that may serve as a regulator of a biological process. The upper molecular weight limit for a small molecule is approximately 900 Daltons which allows for the possibility to rapidly diffuse across cell membranes so that they can reach intracellular sites of action [3, 4]. Small molecules may also be used as research tools to probe biological function as well as leads in the development of new therapeutic agents. Some can inhibit a specific function of a multifunctional protein or disrupt protein-protein interactions [5].

It is well known that the cost of developing a new medicine, that is, new chemical entity, is enormous and takes many years to develop the same due to prolonged biological safety studies and human clinical trials. It also takes a lot of research and development efforts to develop multistep synthesis process and scale up of complex molecules. The number of chiral centres in a molecule also increases its cost to develop and time to market. Hence, new cost-effective, shorter routes of synthesis and relatively small molecules are a need of hour in new chemical entity research [6].

The antimicrobial properties of metals have been recognised for centuries and have represented some of the most fundamental breakthroughs in medicinal history [7]. Several metal complexes are known to accelerate the drug action and efficacy of the organic therapeutic agent. The precious metals platinum and silver were reported to exert a toxic effect on bacteria [8, 9]. The original observations that platinum-ammine complexes had antibacterial properties led to the discovery of their antitumour properties and the development of the highly successful platinum anticancer drugs cisplatin and carboplatin [10]. Complexes of gold have also been reported to have a wide range of antimicrobial activities [11].

In order to begin our efforts for such new medicines as effective anti-infective agents against bacteria and fungi, we thought of combining heterocyclic aniline scaffold with simple ortho hydroxy benzaldehydes like salicylaldehyde to get a Schiff base and its conversion to transition metal complex like Zn(II), Cu(II), Ni(II), and Co(II). In our initial efforts, to screen compounds derived from coumarin scaffold and aliphatic diamino compound like N,N-dimethyl ethylene diamine, we got encouraging results with respect to biological assays against Gram positive bacteria and fungi [12–14].

This diverted our focus to search for new molecular structures having less complex structure and few synthesis steps. We thought of heterocyclic aniline scaffolds and condensed with salicylaldehyde to get corresponding Schiff base and then complexation with zinc metal. Schiff bases were synthesised, isolated, and characterised. Zn(II) complexes were prepared by template method and characterised. Schiff bases and their corresponding Zn(II) metal complexes were evaluated for antibacterial and antifungal activities by MIC method.

## 2. Experimental

All chemicals and solvents used in this work were of analytical grade. Salicylaldehyde was purchased from Merck Chemicals. Zinc chloride, DMSO, and oxalic acid were purchased from SD Fine chemicals.

7-hydroxy-4-methyl-2-oxo-2*H*-chromene-8-carbaldehyde was obtained by Duff formylation procedure starting with 7-hydroxy-4-methyl-2*H*-chromen-2-one as per the reported method [15–17] and N-methyl propane-1,3-diamine was procured from a commercial source.

### 2.1. Preparation of the Schiff Base 7-Hydroxy-4-methyl-8-[(*Z*)-{[3-(methylamino)propyl]imino}methyl]-2*H*-chromen-2-one [NMAPIMHMC] Oxalate Salt (Scheme 1)

The Schiff base, that is, the ligand 8-[(*Z*)-{[3-(N-methylamino)propyl]imino}methyl]-7-hydroxy-4-methyl-2*H*-chromen-2-one [NMAPIMHMC], was synthesized by the condensation of 7-hydroxy-4-methyl-2-oxo-2*H*-chromene-8-carbaldehyde with N-methyl propane-1,3-diamine in (1 : 1) molar proportion in ethanol in the presence of traces of concentrated hydrochloric acid. The reaction mixture was refluxed for an hour. On cooling, the product was isolated as yellowish brown oily mass.

As the oily Schiff base was unstable in nature, it was difficult to characterize the compound. Therefore, its oxalate salt was prepared for spectral characterization.

4-Methyl-7-hydroxy 8-formyl coumarin (1.0 g, 0.0049 mole) was dissolved in 10 mL ethanol and N-methylpropane-1,3-diamine (0.431 g, 0.0049 mole) was added. A drop of concentrated hydrochloric acid was added and the reaction mixture was refluxed for an hour. Oxalic acid (0.555 g, 0.0041 mole, 0.9 eq.) was added and further refluxed for an hour. On cooling, the product was isolated as oxalate salt which was recrystallized from alcohol. The product was filtered and dried in oven till constant weight. Weight: 1.1 g, (yield: 70%). Colour: yellow, M.P. 205–207°C, elemental analysis observed (calculated): C 56.4% (56.02%), H 5.53% (5.69%), N 7.69% (7.22%), UV: *λ*_max⁡_ 225 nm, 313 nm, MS: [M+H]^+^ 275, IR (KBr)  *ν*_N–H_ 3468 cm^−1^ *ν*_C=O (Lacton)_ 1715 cm^−1^ *ν*_C=N_ 1609 cm^−1^ *ν*_C–O–C_ 1076 cm^−1^ *ν*_C–O  (phenolic)_ 1313 cm^−1^, ^1^H NMR [DMSO(d_6_), 300 MHz] 1.92 (s, 3H), 2.2–2.4 (m, 2H), 2.51 (s, 3H), 2.79 (m, 2H), 3.05 (t, 2H), 5.24 (s, 1H), 5.87 (d, 1H, *J* = 9.4 Hz), 6.84 (d, 1H, *J* = 9.4 Hz) 8.15 (s, 1H azomethine).

### 2.2. Preparation of the Zn(II) Complex of “*In Situ*” Schiff Base 7-Hydroxy-4-methyl-8-[(*Z*)-{[3-(methylamino)propyl]imino}methyl]-2*H*-chromen-2-one [NMAPIMHMC] (Scheme 2)

The preparation of the Zn(II) complex was carried out by taking 7-hydroxy-4-methyl-2-oxo-2*H*-chromene-8-carbaldehyde (1.5 g, 0.00735 mol) in ethanol (30 mL) and N-methylpropane-1,3-diamine (0.646 g, 0.00735 mol). A drop of diluted HCl was added and the mixture was refluxed on a water bath for about an hour. The colour of solution was pale yellow. To this hot solution, zinc chloride (1.0 g, 0.00735 mol) was added. The solution was refluxed for additional three hours and TLC was checked for completion of reaction. The pale yellow precipitate formed was filtered, and washed with ethanol. Product was recrystallised in ethanol at reflux, filtered and dried in oven at 70–80°C till constant weight. (Yield: 2.0 g, 42.0%), M.P. > 260°C, [M]^+^ 612, IR (KBr):  *ν*_O–H (lattice  water)_ 3112 cm^−1^ *ν*_C=O (lactonyl)_ 1727 cm^−1^ *ν*_C=N_ 1631 cm^−1^,  *ν*_C−O (phenolic)_ 1371 cm^−1^,  *ν*_Zn–N_ 543 cm^−1^, *ν*_Zn–O_ 453 cm^−1^, ^1^H NMR (DMSO-d_6_, 300 MHz) 2.06 (m, 2H), 2.34 (s, 3H), 2.96 (t, 2H), 3.73 (t, 2H), 5.98 (s, 1H), 6.56 (d, 2H, *J* = 8.4 Hz), 7.56 (d, *J* = 8.4 Hz) 8.83 (s, 1H azomethine).

### 2.3. Preparation of the Schiff Base 2-[(*E*)-{[4-(1*H*-1,2,4-Triazol-1-ylmethyl)phenyl]imino}methyl]phenol [TMPIMP] (Scheme 3)

4-(1*H*-1,2,4-Triazol-1-ylmethyl)aniline was prepared by known method reported in literature [18] and was characterised by spectroscopic techniques. Salicylaldehyde was purchased from Merck Chemicals.

4-(1*H*-1,2,4-Triazol-1-ylmethyl)aniline (0.3 g, 0.00172 mole) was taken in 10 mL ethanol, salicylaldehyde (0.209 g, 0.00172 mole) was added, and solution was heated to reflux in water for about 2 hours. The orange coloured crystalline product was filtered and washed with ethanol. Product was dried in oven at 70–80°C till constant weight. Weight 0.278 g. Colour: yellow crystalline solid, M.P.: 145°C, IR (KBr): *ν*_C=N_ 1621 cm^−1^, phenolic *ν*_C–O_ 1143 cm^−1^, elemental analysis: observed (calculated): C 69.1% (69.05%), H 5.2% (5.07%), N 20.52% (20.13%), MS: [M+H]^+^ 279, ^1^H NMR CDCl_3_ (400 MHz) 5.40 (s, 2H), 6.97 (m, 1H), 7.04 (m, 1H), 7.28–7.31 (m, 3H), 7.35–7.37 (m, 2H), 7.41–7.43 (m, 2H), 8.01 (s, 1H), 8.12 (s, 1H), 8.63 (s, 1H, –H–C=N, azomethine).

### 2.4. Preparation of the Zn(II) Complex of “*In Situ*” Schiff Base 2-[(*E*)-{[4-(1*H*-1,2,4-Triazol-1-ylmethyl)phenyl]imino}methyl]phenol [TMPIMP]

4-(1*H*-1,2,4-triazol-1-ylmethyl) aniline (0.5 g, 0.00287 mol) was dissolved in ethanol (10 mL) and salicylaldehyde (0.350 g, 0.00287 mol) was added. Reaction mass colour changed to yellow. It was heated to reflux and zinc chloride (0.391 g, 0.00287 mole) added and further heated at reflux for 3 hours. Yellow precipitate of the product was filtered and washed with ethanol. It was recrystalised in ethanol and dried in oven at 70–80°C till constant weight. wt: 0.950 g (Yield: 53.3%), Colour: Yellow, M.P. 218-219°C, [M]^+^ 619, IR (KBr): *ν*_O–H  (lattice  water)_ 3312 cm^−1^ *ν*_C=N  _ 1619 cm^−1^, *ν*_C–O  (phenolic)_ 1452 cm^−1^, *ν*_Zn–N  _ 522 cm^−1^, *ν*_Zn–O_ 447 cm^−1^, ^1^H NMR (DMSO-d_6_, 300 MHz) 5.48 (s, 2H), 6.96–7.0 (m, 3H), 7.42 (m, 5H), 8.04 (s, 1H), 8.8 (s, 1H), 9.03 (s, 1H, –H–C=N, azomethine).

### 2.5. Preparation of the Schiff Base (4*S*)-4-{4-[(*E*)-(2-Hydroxybenzylidene)amino]benzyl}-1,3-oxazolidin-2-one [HBABO] (Scheme 4)

(4*S*)-4-(4-Aminobenzyl)-1,3-oxazolidin-2-one was synthesised by reported method in literature [19] and was characterised by spectroscopic techniques.

(4*S*)-4-(4-Aminobenzyl)-1,3-oxazolidin-2-one (0.3 g, 0.00156 mole) was taken in 10 mL ethanol, salicylaldehyde (0.190 g, 0.00156 mole) was added, and solution was heated to reflux for about 2 hours. The light orange coloured product was filtered, washed with ethanol, and again recrystallised in ethanol. Product was dried in oven at 70–80°C till constant weight. Weight 0.40 g. Colour: light orange, M.P. 188–190°C, IR (KBr): *ν*_N–H_ 3362 cm^−1^ *ν*_C=O  (oxazolidone)_ 1752 cm^−1^ *ν*_C=N_ 1644 cm^−1^ *ν*_C–O–C_ 1074 cm^−1^ *ν*_C−O  (phenolic)_ 1147 cm^−1^, elemental analysis: observed (calculated): C 68.72% (68.91%), H 5.49% (5.44%), N 9.52% (9.45%), MS: [M+H]^+^ 297.4.

### 2.6. Preparation of the Zn(II) Complex of “*In Situ*” Schiff Base (4*S*)-4-{4-[(*E*)-(2-Hydroxybenzylidene)amino]benzyl}-1,3-oxazolidin-2-one [HBABO]

(4*S*)-4-(4-Aminobenzyl)-1,3-oxazolidin-2-one (0.3 g, 0.00156 mole) was dissolved in 10 mL Ethanol, salicylaldehyde (0.190 g, 0.00156 mole) was added, solution was heated to for about 30 minutes, and zinc chloride (0.212 g, 0.00156 mol) was added and further heated for 3 hours. Yellow coloured product was filtered, washed with ethanol, and recrystallised in ethanol and dried in oven till constant weight. Weight 0.80 g (yield: 78.43%), colour: yellow, M.P. 240°C (decomp.), MS: [M+1] 657.

IR (KBr): *ν*_O–H  (lattice  water)_ 3282 cm^−1^ *ν*_C=O  (oxazolidone)_ 1750 cm^−1^ *ν*_C=N  _1625 cm^−1^, *ν*_C–O  (phenolic)_ 1446 cm^−1^, *ν*_Zn–N_ 530 cm^−1^,  *ν*_Zn–O_ 449 cm^−1^, elemental analysis: observed (calculated): C 59.83% (59.01), H 4.81% (4.95%), N 8.15% (8.10%), ^1^H NMR (DMSO-d_6_, 300 MHz) 2.47–2.81 (m, 2H), 3.97–4.06 (m, 2H), 4.23–4.28 (m, 1H), 6.92–6.98 (m, 2H), 7.33–7.38 (m, 4H), 7.61 (d, 2H, *J* = 7.2 Hz), 7.81 (s, 1H N–H oxazolidinone), 8.94 (bs, 1H azomethine).

^13^C NMR (DMSO-d_6_, 75 MHz) 163.04 (–C=N azomethine), 160.26 (oxazolidone –C=O), 158.60 (–C–O phenolic), 146.43, 135.48, 133.18, 132.53, 130.49, 121.36, 119.10, 116.55, 67.97, 52.42, 39.90.

## 3. Results and Discussion

All the metal complexes are stable at room temperature and are nonhygroscopic in nature. On heating, they decompose at high temperatures. The complexes are insoluble in water but are soluble in DMSO. The elemental analysis, physical properties, and analytical data of the ligand and complexes are summarized below.

### 3.1. ^1^H NMR Spectra

Due to the diamagnetic nature of Zn(II) metal complexes, it was possible to scan ^1^H NMR spectrum in DMSO-d_6_. Diamagnetic zinc metal complexes do not interfere in magnetic field of NMR instrument; however, paramagnetic metal complexes interfere and it is not possible to lock NMR instrument for scanning samples.

It was observed that the azomethine proton in [Zn(NMAPIMMTC)_2_]·2H_2_O complex appeared at 8.83 ppm after complexation with zinc metal. It was shifted significantly downfield due to deshielding effect exerted by zinc metal atom. Apart from the downfield shift of azomethine, following other interesting observations were also made. Aromatic protons of coumarin ring were observed at 6.56 ppm and 7.56 ppm as doublets due to the electron withdrawing mesomeric effect exerted by central zinc metal atom. Olefinic proton of coumarin ring was also shifted downfield to 5.98 ppm due to electron withdrawing mesomeric effect operating through the conjugation across the aromatic ring over the *α*,*β*-unsaturated double bond of coumarin ring.

The azomethine proton in [Zn(TMPIMP)_2_]·2H_2_O complex was observed at 8.63 ppm and that of [Zn(HBABO)_2_]·2H_2_O was observed at 8.94 ppm.

**Figure 1:**
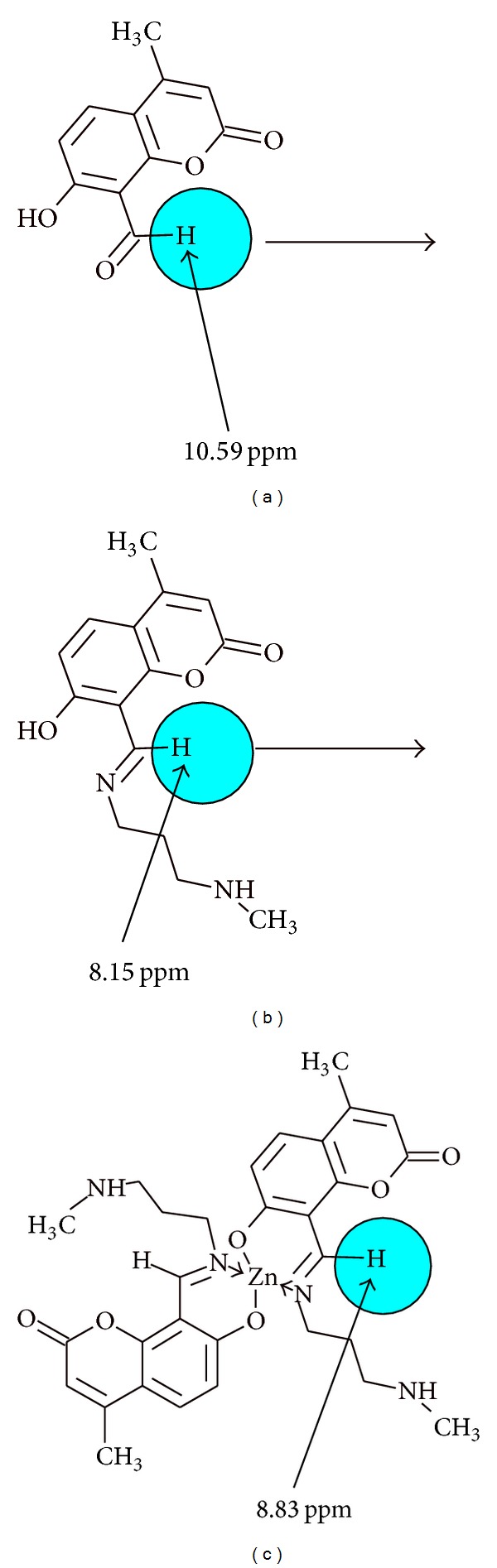
Azomethine proton shift values before and after complexation with Zn(II) metal atom.

Thus, the azomethine H–C=N protons which appeared at about 8.83 to 9.12 in free Schiff bases were shifted to downfield to 8.63 to 8.94 due to electron withdrawing effect of central metal atom (see Figure 1). The integration of all the proton indicated Zn : (L)_2_ stoichiomery of the complexes.

### 3.2. ^13^C NMR Spectra

The azomethine carbon atom appeared most downfield as reported in literature values. In [Zn(NMAPIMMTC)_2_]·2H_2_O complex, it was observed at 162.73 ppm, in [Zn(HBABO)_2_]·2H_2_O complex, it was observed at 163.04 ppm, and, in [Zn(TMPIMP)_2_]·2H_2_O complex, it appeared at 163.70 ppm (Table 1).

Normally carbon attached to phenolic –OH group appears at about 155 ppm, but, in these complexes, it was observed at 155–160 ppm which may be due to electron deshielding effect of zinc metal atom.

In [Zn(NMAPIMMTC)_2_]·2H_2_O, lactonyl carbon appeared at 173.63 ppm, and, in [Zn(HBABO)_2_]·2H_2_O complex, the oxazolidinone carbonyl carbon appeared at 160.26 ppm.

### 3.3. Mass Spectra

The formation of Schiff bases is confirmed by the presence of intense molecular ion peak in the mass spectra of Schiff base metal complexes such as [Zn(NMAPIMMTC)_2_]·2H_2_O, [Zn(HBABO)_2_]·2H_2_O, and [Zn(TMPIMP)_2_]·2H_2_O. Other prominent peaks may be due to the elimination of CH_3_NH, –CH_2_–CH_2_–CH_2_– units of propyl side chain in case of [Zn(NMAPIMMTC)_2_]·2H_2_O. In other complexes, prominent peaks may be due to the fragmentation of heterocyclic rings in the molecules.

Some other peaks may be due to loss of tropylium ion and so forth from the parent ion and subsequent fragmentation. The mass spectra of the Zn(II) complexes showed molecular ion peaks corresponding to [M(L)_2_] stoichiometry. Peaks corresponding to L+ and fragments of L+ are also present in the spectra. Detection of [M^+^] and [M+1]^+^ peaks in mass spectra indicated and confirmed Zn : (L)_2_ stoichiometry of the complexes.

#### 3.3.1. Infrared Spectra

The interpretation of IR spectra provides valuable information regarding the nature of functional group attached to the metal atom and helped in confirmation of bond formation. In order to study the bonding mode of Schiff base ligand to the central metal atom, the IR spectra of the free ligands were compared with the spectra of the complexes. The main IR bands and their assignments are listed in Table 2.

The Schiff base HBABO has an oxazolidinone –N–C=O functional group and it has been observed as carbonyl stretching band at 1737 cm^−1^ in [Cu(HBABO)_2_]·2H_2_O complex and at 1750 cm^−1^ in [Zn(HBABO)_2_]·2H_2_O complex. All the above metal complexes have also shown absorption bands in the region 3400–3500 cm^−1^ due to coordinated water molecules Table 2.

- The phenolic –OH band does not appear in metal complexes spectra. However new bands have appeared at 1621 cm^−1^ to 1639 cm^−1^ due to new –C=N, that is, azomethine double bond, which is characteristic of Schiff base and confirms the formation of Schiff bases and further complexation with central metal atom.
- The IR spectra of all the metal complexes show prominent band at about 1240–1280 cm^−1^ due to *ν*_C–N  _ stretching.
- There are no prominent bands appearing in the 1600–1800 cm^−1^ region of the spectra indicating participation of the azomethine nitrogen and phenolic oxygen atom in coordination with the metal atom [20].
- The broad signals in the region of 2500 cm^−1^ to 3500 cm^−1^ of the Schiff base ligands disappeared in the spectra of all the metal complexes indicating complexation with central metal cation. However, the spectra of the metal complexes in this region show a number of signals arising from *ν*_C–H_ and vibrations due to coordinated H_2_O molecules.
- The low frequency region of the spectra indicated the presence of two new medium intensity bands at about 450 cm^−1^ to 470 cm^−1^ due to *ν*_M–O_ vibrations and at 530 cm^−1^ to 550 cm^−1^ due to *ν*_M–N_ vibrations [21].

#### 3.3.2. Thermogravimetric Analysis

Thermogravimetric analysis showed a loss of about 5.5% in weight corresponding to weight of two water molecules from the compound. This is water coordinated to central metal atom. Further heating resulted in continuous loss in weight with rise in temperature indicating decomposition of samples above 250°C.

It is clear from the data presented above that the experimental values of each compound are in good agreement with the theoretical values calculated for 1 : 2 ratio of metal : ligand stoichiometry. This is confirmed by M+ and [M+1] peaks in high resolution mass spectra.

From the discussion of the results of various spectroscopic details presented above, it may be concluded that the proposed geometry for the transition metal complexes with general formula ML_2_·2H_2_O is octahedral for Zn(II) complexes. The probable structures are shown in Table 3.

### 3.4. Biological Assay

#### 3.4.1. Antibacterial Studies

Antimicrobial activity of the Schiff base ligand and its metal complexes was screened against two Gram negative bacteria: *E. coli* and *Pseudomonas fluorescens*, one Gram positive bacteria: *Staphylococcus aureus,* and against two fungi, that is, *C. albicans* and *A. niger* to assess their potential as antimicrobial agent by MIC method.

#### 3.4.2. Microbiological Method [22]: MIC Procedure (for Bacteria and Fungus)

The following ATCC strains were procured from Institute of Microbial Technology, Chandigarh, India: *E. coli*: ATCC no. 25922, *P. aeruginosa*: ATCC no. 25619, *S. aureus*: ATCC no. 12598, *Candida albicans*: ATCC no. 2091, *Aspergillus niger*: ATCC no. 9029.

Inoculum used was matched with 0.5 Mac Farland standard, that is, equal to 3 × 10^5^ CFU/mL.

DMSO was used as solvent control. The solvent DMSO had no antimicrobial effect at the concentrations employed. DMSO used was commercially available. 10 mg of the test compound was dissolved in 1 mL of DMSO and this solution was used as stock solution for the test.

Nine dilutions of each drug were done with brain heart infusion (BHI) for MIC. In the initial tube, 20 *μ*L of above drug stock solution (10 mg/mL) was added into the 380 *μ*L of BHI broth. For dilutions, 200 *μ*L of BHI broth was added into the next 9 tubes separately. Then, from the initial tube, 200 *μ*L was transferred to the first tube containing 200 *μ*L of BHI broth. This was considered as 1 × 10^−1^ dilution. From 1 × 10^−1^ diluted tube, 200 *μ*L was transferred to second tube to make 1 × 10^−2^ dilution. The serial dilution was repeated up to 1 × 10^−9^ dilution for each drug. From the maintained stock cultures of required organisms, 5 *μ*L was taken and added into 2 mL of BHI (brain heart infusion) broth. In each serially diluted tube, 200 *μ*L of above culture suspension was added. The tubes were incubated for 24 hours at 37°C in the incubator and observed for turbidity. (Note: for facultative anaerobes, tubes were incubated at 37°C for 48–72 hrs in CO_2_ Jar.)

Ciprofloxacin and Fluconazole were used as standards. Microbroth dilution method was used for the standard drugs.

Antifungal activity was carried out in a biosafety cabinet to avoid the contamination.

### 3.5. *In Vitro* Antimicrobial Activity

A comparative study of MIC values of Schiff base and its complexes indicated that metal complexes exhibit higher antimicrobial activity than the free Schiff base ligands and the same is indicated from the results given in Table 4.

There was no promising antibacterial activity observed against Gram negative bacteria, that is, *E. coli* and *Pseudomonas*. It was in the range of MIC value of 50–100 *μ*g/mL concentration compared to standard antibiotic Ciprofloxacin having MIC of 2 *μ*g/mL. This may be due to effective barrier of an outer membrane of Gram negative bacteria, towards intake of external substances like test compounds under this study.

The sensitivity of the test organisms to the test compounds may also be associated with cell wall structure. The major role of action involves highly specific coordination of metal ion to thiol groups on proteins containing L-cysteine [7]. The reduced activity of the test compounds may be due to lack of such coordination of Zn(II) to form a specific complex with cell wall protein thiol groups.

However, in case of *S. aureus*, [Zn(NMAPIMHMC)_2_]·2H_2_O complex showed moderate activity up to MIC value of 12.5 *μ*g/mL and [Zn(TMPIMP)_2_]·2H_2_O complex showed activity up to MIC value of 6.25 *μ*g/mL. This could be due to coordination of Zn(II) atom to form a specific complex with cell wall protein thiol groups and ultimately interfering in cell wall synthesis of *S. aureus* during cell multiplication phase. The observed activity of the test compounds indicates the future potential for the development of metal coordination complexes to overcome the limitations due to currently available antibiotics to treat MSRA.

In case of antifungal activity against *C. albicans*, [Zn(NMAPIMHMC)_2_]·2H_2_O and [Zn(TMPIMP)_2_]·2H_2_O complexes showed most promising activity up to MIC value of 3.12 *μ*g/mL, compared to standard Fluconazole having MIC value 16 *μ*g/mL.

However, in case of antifungal activity against *A. niger*, Zn(NMAPIMHMC)_2_]·2H_2_O showed the most promising activity up to MIC value of 0.8 *μ*g/mL compared with standard Fluconazole having MIC value of 8 *μ*g/mL. [Zn(TMPIMP)_2_]·2H_2_O and [Zn(HBABO)_2_]·2H_2_O complexes also showed better activity up to MIC value of 3.12 *μ*g/mL.

In almost all the comparative studies done, metal complexes showed enhanced activity compared with Schiff base ligand. These observations are due to heterocyclic rings of coumarin moiety, triazole heterocyclic ring, and oxathiazolidinone heterocyclic ring incorporated in the molecular structure of the metal complexes. These structural scaffolds might interfere in the mechanism of cell multiplication as discussed above and hence stop further growth of fungus.

It is known that chelation tends to make the ligand act as more powerful and potent bacterial agent. This may be probably due to the greater lipophilic nature of the complexes. Such increased activity of the metal chelates can be explained on the basis of chelation theory [23]. As per Overtone's concept of cell permeability, the lipid membrane that surrounds the cell favours the passage of only lipid soluble materials. Liposolubility of a molecule is an important factor which controls the antimicrobial activity. On chelation, the polarity of the metal ion is reduced to a greater extent due to overlap of the ligand orbital and partial sharing of positive charge of metal ion with donor groups of the Schiff base ligand [24, 25]. As a result, due to increased delocalization of the *π* electrons over the whole chelate ring, the lipophilicity of the complex is increased. This increased lipophilicity facilitates the penetration of the complexes into lipid membrane and then blocks the metal binding sites on enzymes of microorganisms [26]. These metal complexes also disturb the respiration process of the cell and thus block the synthesis of proteins. As synthesis of proteins is blocked, bacterial cell wall formation is not possible; hence, it results in cell death and ultimately restricts further growth of the organism [27].

According to yet another plausible mechanism, these complexes might be inhibiting DNA gyrase enzyme, which is responsible for DNA multiplication phases. Since DNA gyrase is inhibited by metal complexes, multiplication of bacterial cells is stopped, ultimately resulting in antibacterial activity [7, 28, 29].

## 4. Conclusion

Three novel Schiff bases 8-[(*Z*)-{[3-(N-methylamino)propyl]imino}methyl]-7-hydroxy-4-methyl-2*H*-chromen-2-one, 2-[(*E*)-{[4-(1*H*-1,2,4-triazol-1-ylmethyl)phenyl]imino}methyl]phenol, and (4*S*)-4-{4-[(*E*)-(2-hydroxybenzylidene)amino]benzyl}-1,3-oxazolidin-2-one have been synthesised and structurally characterised. Novel transition metal complexes derived from these Schiff bases have been synthesised and characterised by spectroscopic techniques.

The physical and spectral analytical data show that the metal ligand stoichiometry in all these complexes is 1 : 2. The spectral data show that the ligand is bidentate which coordinates through the azomethine nitrogen of Schiff base ligand and oxygen atom of salicylaldehyde fragment. Based on analytical and spectral data, all these complexes are assigned to be in octahedral geometry.

Some of the Zn(II) metal complexes have shown significant antifungal activities compared to its Schiff base ligand and moderate antibacterial activity. Schiff base Zn(II) metal coordination complexes can be used not only as an approach to enhance their activity but also to overcome the drug resistance.

In conclusion, the “*in vitro*” data presented here has identified Zn(NMAPIMHMC)_2_]·2H_2_O, [Zn(TMPIMP)_2_]·2H_2_O, and [Zn(HBABO)_2_]·2H_2_O as a metal containing complex of potential therapeutic benefit, particularly for the topical treatment as antifungal agent against *C. albicans* and *A. niger*.

## Supplementary Material

Supplementary material consisting of ^1^HNMR and ^13^CNMR spectra including DEPT are included. These were recorded on Bruker 300 MHz instrument. A high-resolution mass spectrometer, API4000 Triple quadrupole mass spectrometer (AB Sciex instruments, Canada) was used for the ionization and fragmentation study of metal complexes. This instrument consists of a quadrupole mass analyzer followed by Qtrap. IR spectra were recorded in KBr pellet using Shimadzu FTIR instrument. TGA graphs were recorded using Metler Toledo instrument.

## Figures and Tables

**Scheme 1:**
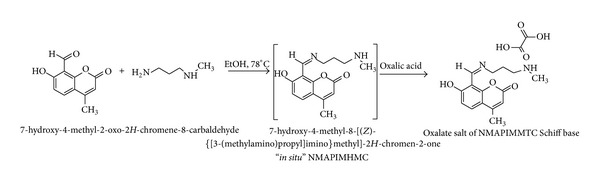
Synthesis of Schiff base 7-hydroxy-4-methyl-8-[(*Z*)-{[3-(methylamino)propyl]imino}methyl]-2*H*-chromen-2-one [NMAPIMHMC] oxalate salt.

**Scheme 2:**
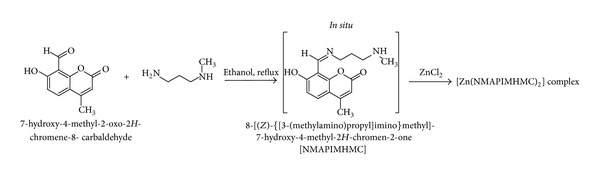
Synthesis of Zn(II) complex of Schiff base 7-hydroxy-4-methyl-8-[(*Z*)-{[3-(methylamino)propyl]imino}methyl]-2*H*-chromen-2-one [NMAPIMHMC].

**Scheme 3:**
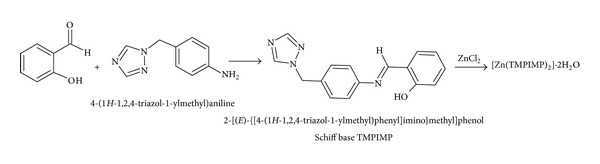
Synthesis scheme of Schiff base TMPIMP and complex [Zn(TMPIMP)_2_]·2H_2_O.

**Scheme 4:**
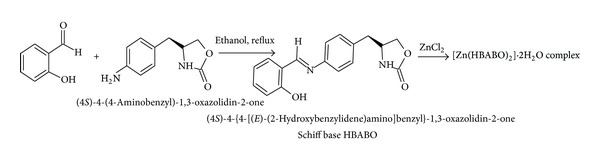
Synthesis scheme of Schiff base ligand HBABO and complex [Zn (HBABO)_2_]·2H_2_O.

**Table 1:** ^13^C NMR assignments of metal complexes.

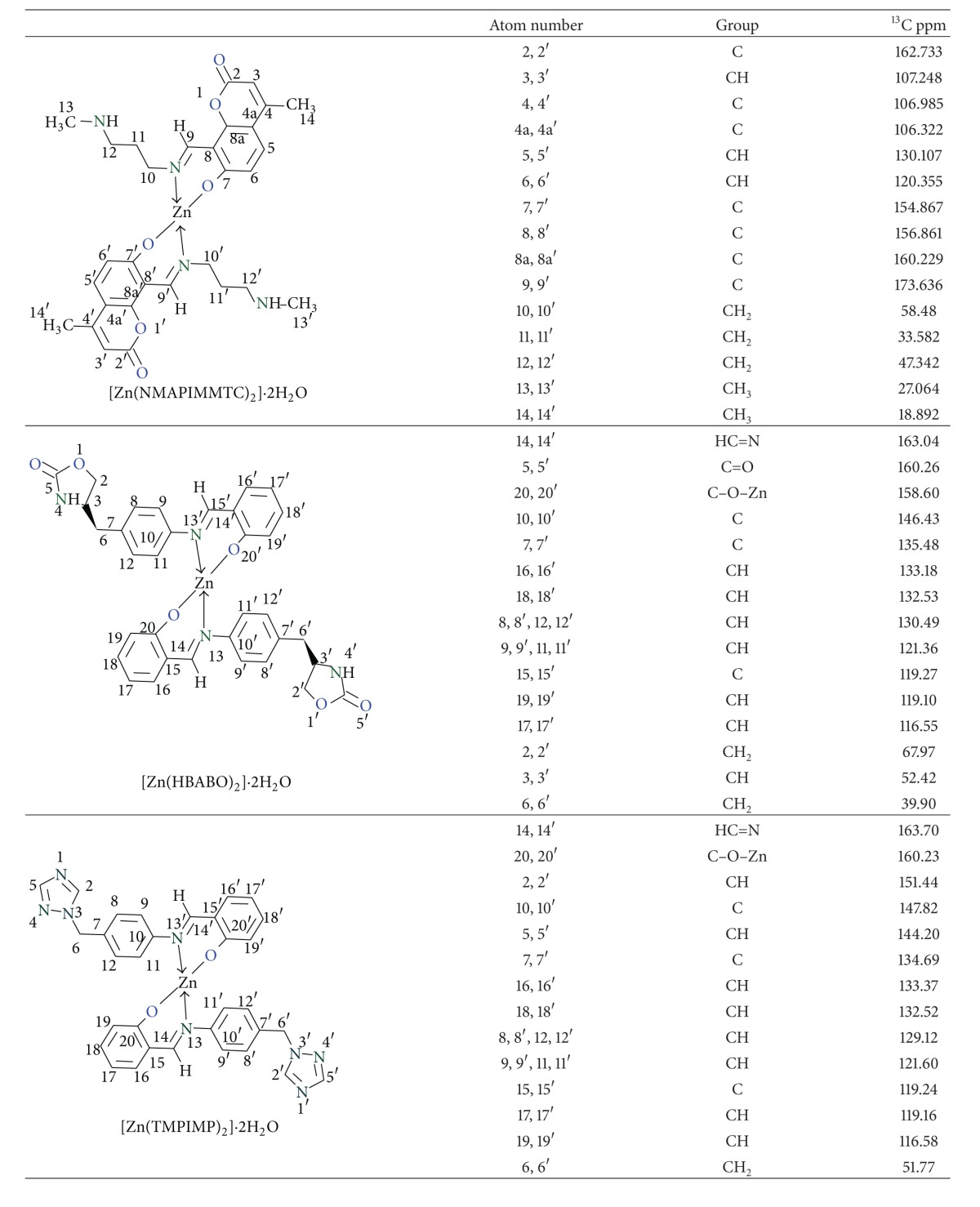

**Table 2:** FT-IR bands for metal complexes and their assignments.

Complex	Lattice water *ν*_(OH)_ cm^−1^	*ν*_C=O_ cm^−1^	*ν*_C=N_ cm^−1^	Phenolic_C–O_ cm^−1^	*ν*_M–N_ cm^−1^	*ν*_M–O_ cm^−1^
[Zn(NMAPIMHMC)_2_]·2H_2_O	3122	1727 (lactone)	1631	1371	543	453
[Zn(HBABO)_2_]·2H_2_O	3282	1750 (Oxazolidinone)	1625	1446	530	449
[Zn(TMPIMP)_2_]·2H_2_O	3312	NA	1619	1452	522	447

**Table 3:** Proposed structure of [Zn(L)_2_]·2H_2_O complexes.

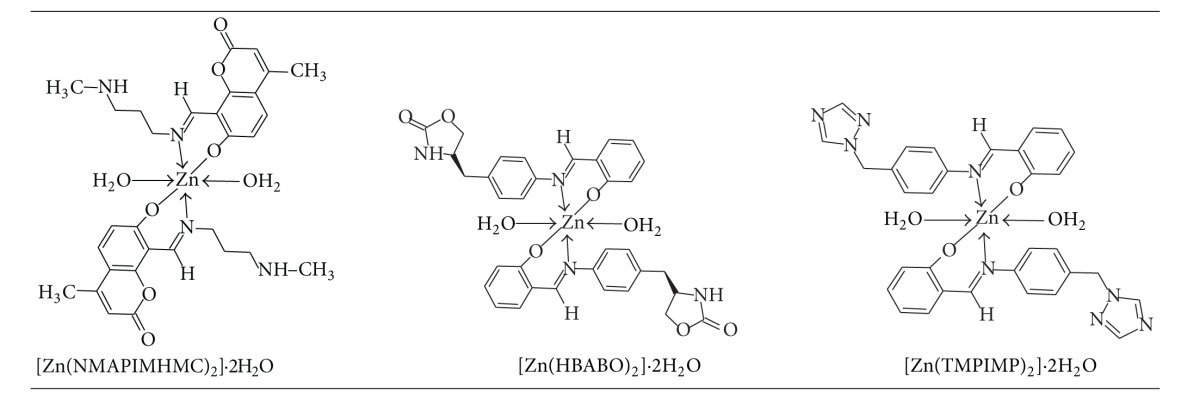

**Table 4:** Showing comparative antibacterial and antifungal screening results by MIC method.

Test compounds	Test organism and sample concentration in *µ*g/mL				
	*E. coli *	*P. aeruginosa *	*S. aureus *	*C. albicans *	*A. niger *
NMAPIMHMC.oxalate	50	50	50	50	0.8
[Zn(NMAPIMHMC)_2_]·2H_2_O	50	50	12.5	3.12	0.8
TMPIMP	100	50	12.5	50	1.6
[Zn(TMPIMP)_2_]·2H_2_O	100	100	6.25	3.12	3.12
HBABO	100	100	6.25	12.5	6.25
[Zn(HBABO)_2_]·2H_2_O	100	100	12.5	25	3.12
Standard Ciprofloxacin	2	<4	2	—	—
Standard Fluconazole	—	—	—	16	8
