# Supplementary material for: Novel Zinc(II) Complexes of Heterocyclic Ligands as Antimicrobial Agents: Synthesis, Characterisation, and Antimicrobial Studies
Source: Bioinorg Chem Appl. 2014 Feb 23;2014:276598. doi: 10.1155/2014/276598 (PMC3953498; doi:10.1155/2014/276598)
Supplement: Supplementary file 1 — Supplementary material consisting of 1HNMR and 13CNMR spectra including DEPT are included. These were recorded on Bruker 300 MHz instrument. A high-resolution mass spectrometer, API4000 Triple quadrupole mass spectrometer (AB Sciex instruments, Canada) was used for the ionization and fragmentation study of metal complexes. This instrument consists of a quadrupole mass analyzer followed by Qtrap. IR spectra were recorded in KBr pellet using Shimadzu FTIR instrument. TGA graphs were recorded using Metler Toledo instrument. [file 276598.f1.pdf]

Supporting information

Novel Zinc (II) complexes of heterocyclic ligands as antimicrobial agents: Synthesis, characterisation and antimicrobial studies

Ramesh S. Yamgar[1], Y. Nivid[2], Satish Nalawade[2], Mustapha Mandewale[2], R. G. Atram[2] and Sudhir S. Sawant[2]*

[1]Department of Chemistry, Chikitsak Samuha's Patkar-Varde College of Arts, Science and Commerce, Goregaon (W), Mumbai 400 062, INDIA.

[2] P. G. Dept of Chemistry, Govt. of Maharashtra, Ismail Yusuf Arts, Science and Commerce College, Jogeshwari (East), Mumbai 400 060 INDIA

***Corresponding author: Tel +91 9963476797 e-mail: sawantsudhir@hotmail.com**

No.	Contents	Pg No.
1	Figure S1 MS Spectrum of NMAPIMHMC Schiff base	3
2	FigureS2 ^{1}H NMR spectrum of [Zn(NMAPIMHMC)$_2$].2H$_2$O	3
3	Figure S3 ^{13}C NMR spectrum of [Zn(NMAPIMHMC)$_2$].2H$_2$O	4
4	Figure S4 ^{13}C NMR DEPT spectrum of [Zn(NMAPIMHMC)$_2$].2H$_2$O	4
5	Figure S5 MS of Zn(NMAPIMHMC)2.2H2O full scan	5
6	Figure S6 MS-MS of Zn(NMAPIMHMC)2.2H$_2$O Parent ion peak	5
7	Figure S7 IR Spectrum of Zn(NMAPIMHMC)$_2$.2H$_2$O	6
8	Figure S8 ^{1}H NMR spectrum of Schiff base TMPIMP	6

9	Figure S9 Mass spectrum of [Zn(TMPIMP)2].$2H_2O$	7
10	Figure S10 ^{1}H NMR spectrum of [Zn$(TMPIMP)_2$].$2H_2O$ SS151	7
11	Figure S11 ^{13}C NMR spectrum of [Zn$(TMPIMP)_2$].$2H_2O$ SS151	8
12	Figure S12 ^{13}C NMR DEPT spectrum of [Zn$(TMPIMP)_2$].$2H_2O$ SS151	9
13	Figure S13 IR spectrum of [Zn$(TMPIMP)_2$].$2H_2O$ SS151	10
14	Figure S14 ^{1}H NMR Spectrum of Schiff base HBABO	10
15	Figure S15 MS Spectrum of HBABO Schiff base	11
16	Figure S16 IR Spectrum of HBABO Schiff base	11
17	Figure S17 ^{1}H NMR Spectrum of Zn$(HBABO)_2$.$2H_2O$	12
18	Figure S18 ^{13}C NMR Spectrum of Zn$(HBABO)_2$.$2H_2O$	13
19	Figure S19 ^{13}C NMR DEPT Spectrum of Zn$(HBABO)_2$.$2H_2O$	14
20	Figure S20 MS Spectrum of Zn$(HBABO)_2$.$2H_2O$	15
21	Figure S21 IR Spectrum of Zn$(HBABO)_2$.$2H_2O$	15
22	Figure S22 TGA graph of Zn$(NMAPIMHMC)_2$.$2H_2O$	16
23	Figure S23 TGA graph of Zn$(TMPIMP)_2$.$2H_2O$	17
24	Figure S24 Antibacterial activity of [Zn$(HBABO)_2$].$2H_2O$ against *Pseudomonas*	17
25	Figure S25 Antibacterial activity of [Zn$(DMAEBIMP)_2$].$2H_2O$ against *Pseudomonas*	18
26	Figure 26 Antibacterial activity of [Zn$(NMAPIMHMC)_2$].$2H_2O$ against *S. aureus*	18
27	Figure S27 Antibacterial activity of [Zn$(HBABO)_2$].$2H_2O$ against *E.Coli*	18

28	Figure S28 Antifungal activity of $[Zn(DMAEBIMP)_2].2H_2O$ against A.niger	19
29	Figure S29 Antifungal activity of $[Zn(HBABO)_2].2H_2O$ against A.niger	19
30	Figure S30 Antifungal activity of $[Zn(DMAPIMMTC)_2].2H_2O$ against *Candida a.*	19

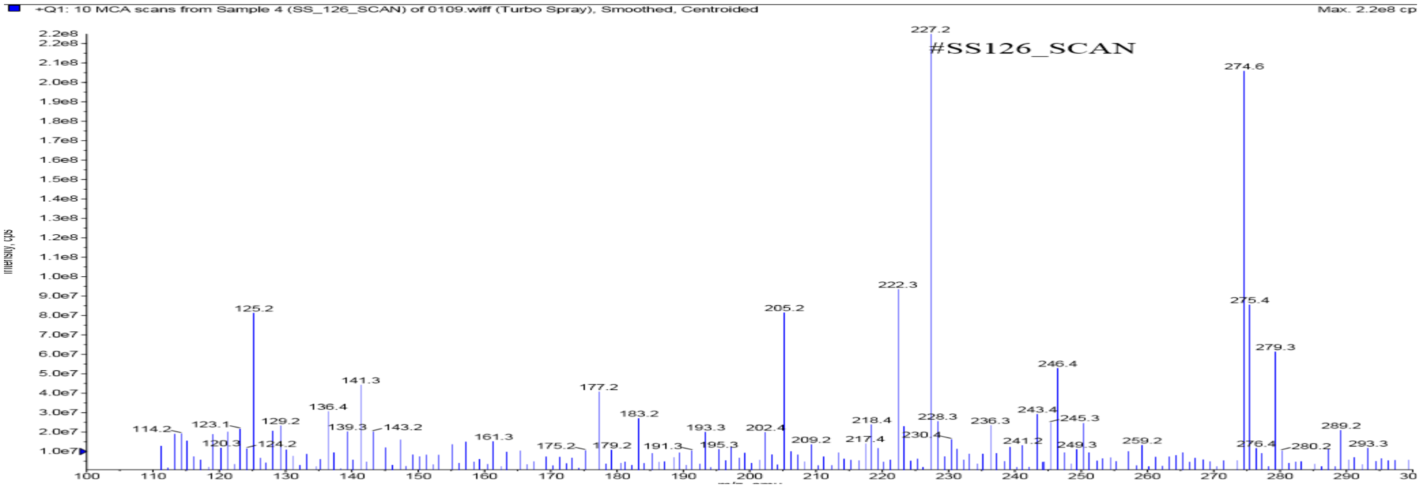

Figure S1 MS Spectrum of NMAPIMHMC Schiff base

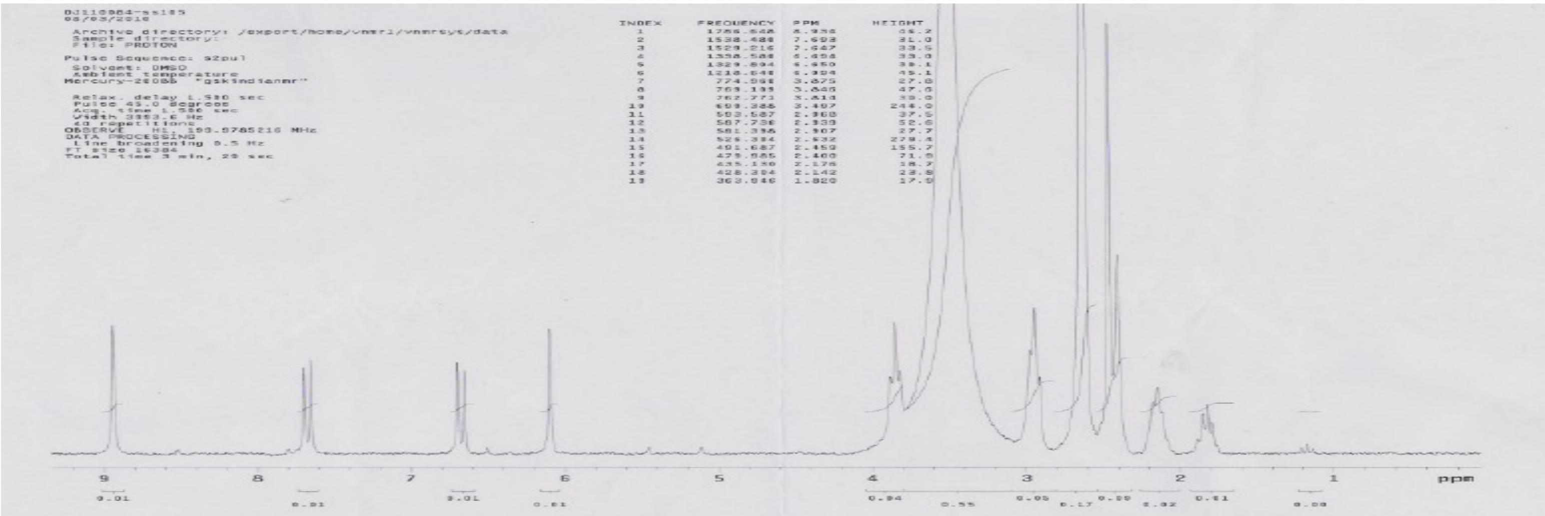

FigureS2 ^{1}H NMR spectrum of $[Zn(NMAPIMHMC)_2].2H_2O$

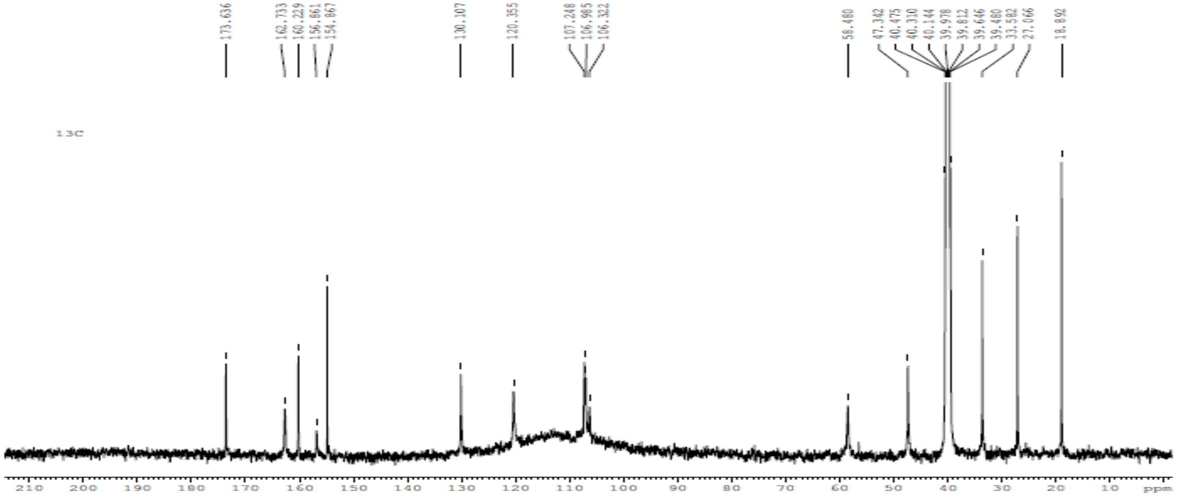

Figure S3 ^{13}C NMR spectrum of $[Zn(NMAPIMHMC)_2].2H_2O$

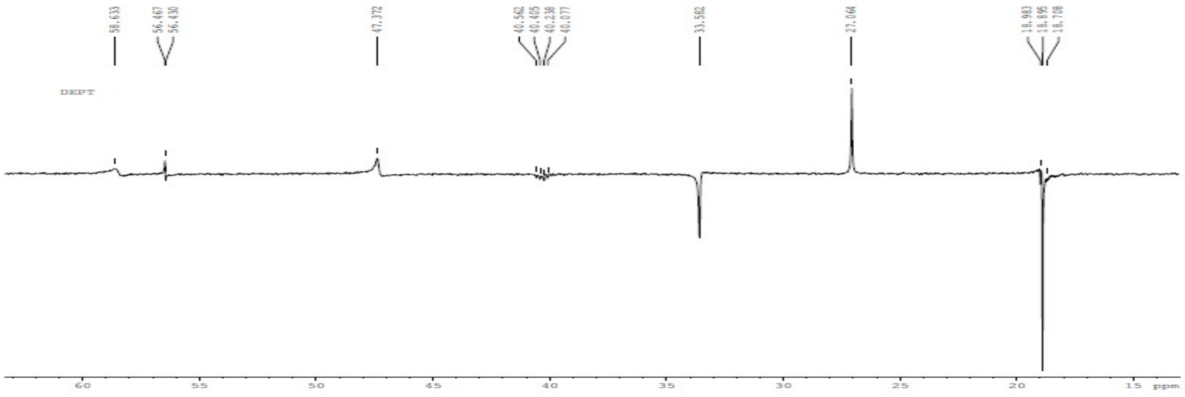

Figure S4 ^{13}C NMR DEPT spectrum of $[Zn(NMAPIMHMC)_2].2H_2O$

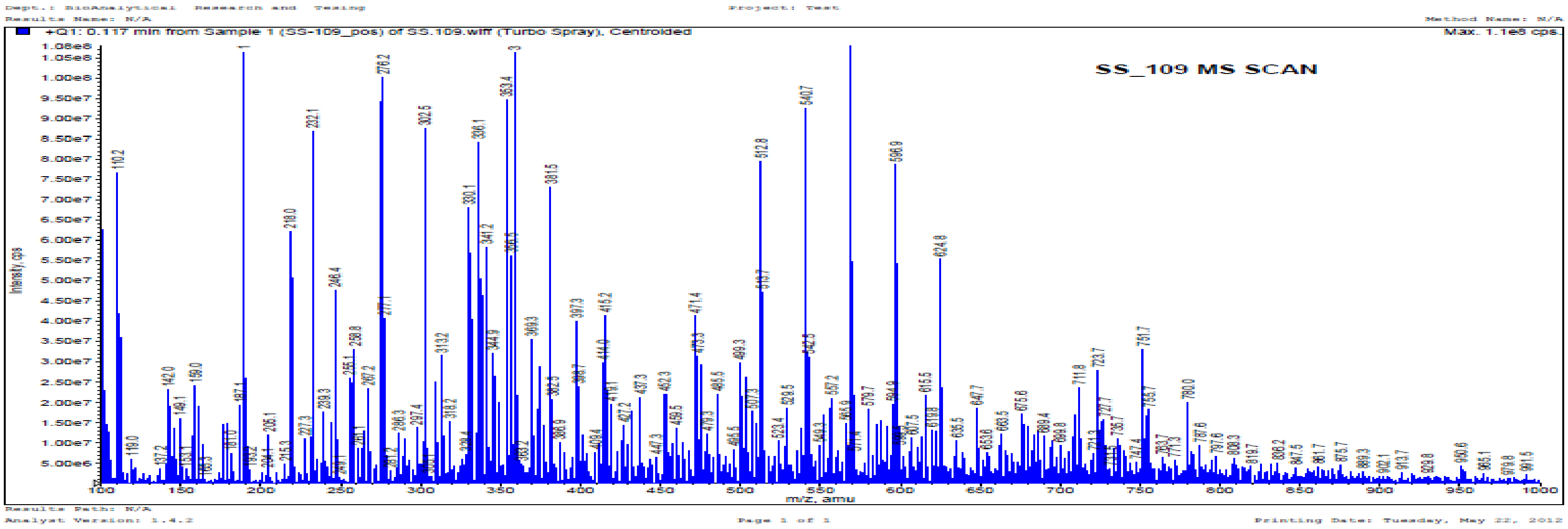

Figure S5 MS of Zn(NMAPIMHMC)2.2H2O full scan

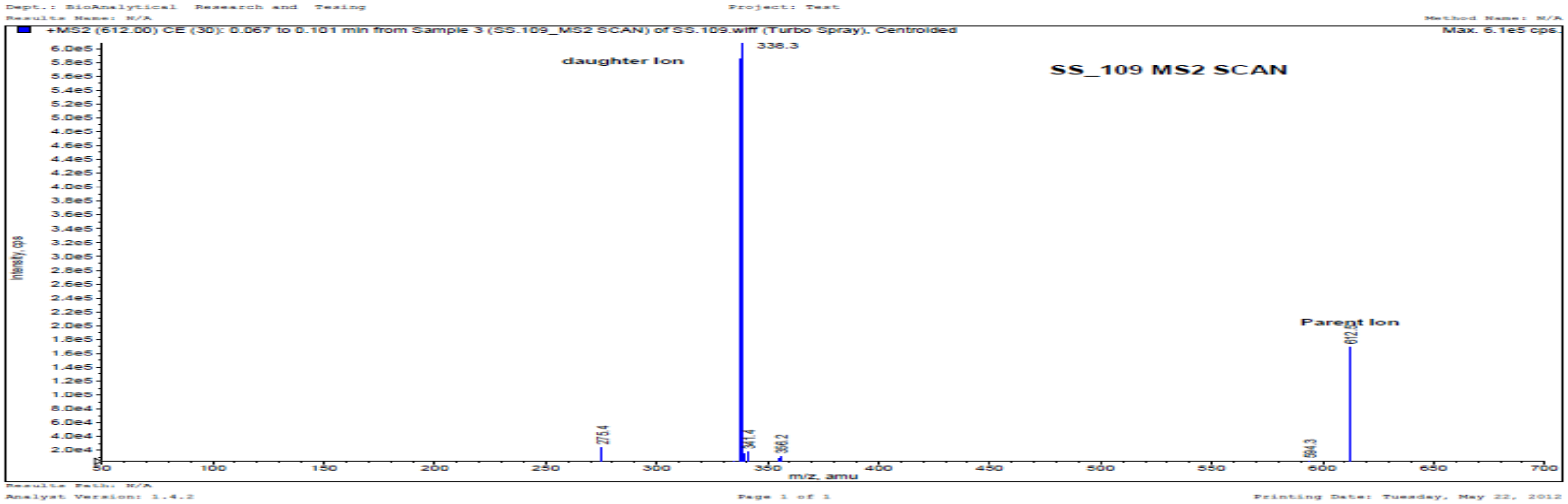

Figure S6 MS-MS of Zn(NMAPIMHMC)2.2H_2O Parent ion peak

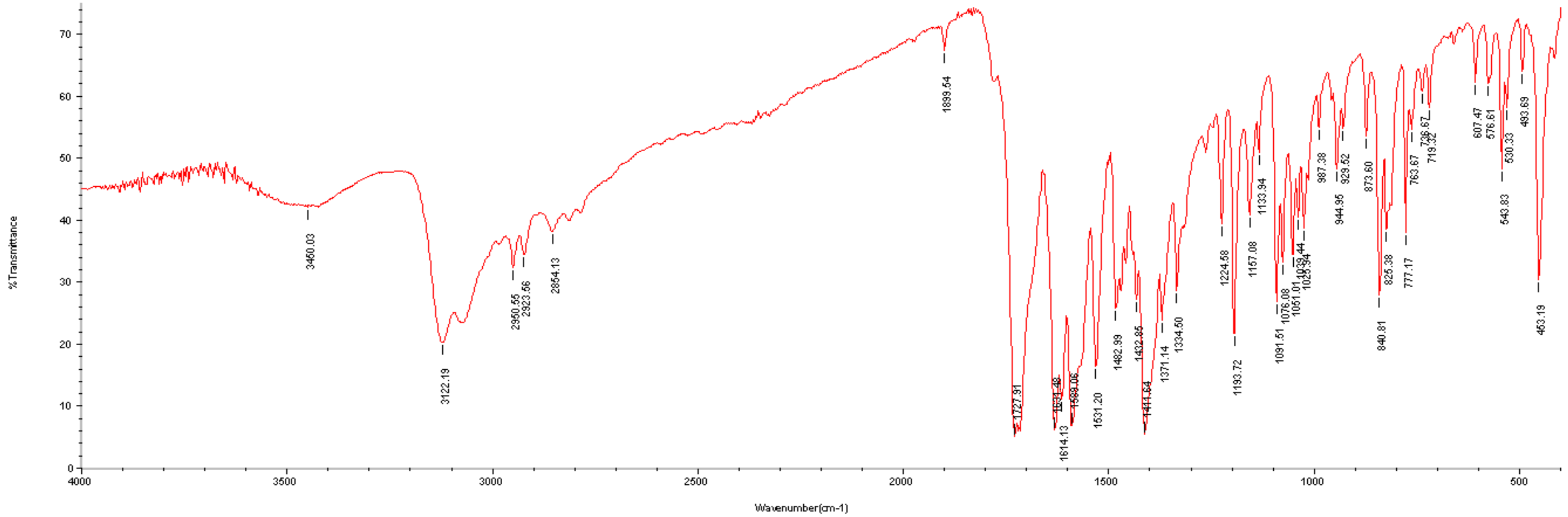

Figure S7 IR Spectrum of Zn(NMAPIMHMC)$_2$.2H_2O

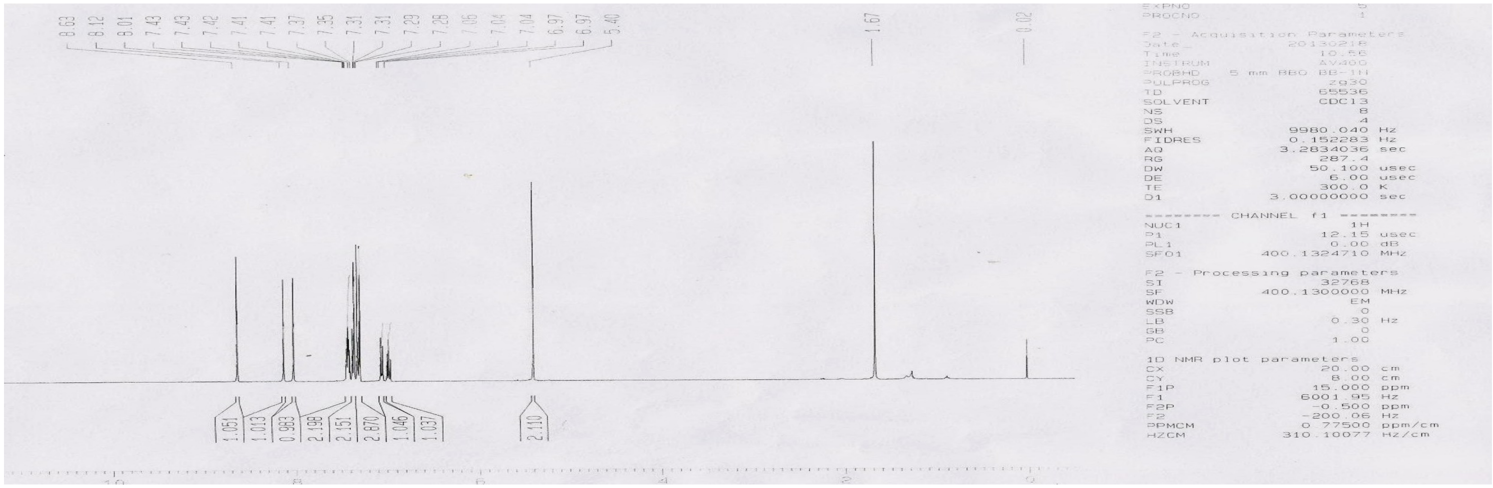

Figure S8 ^{1}H NMR spectrum of Schiff base TMPIMP

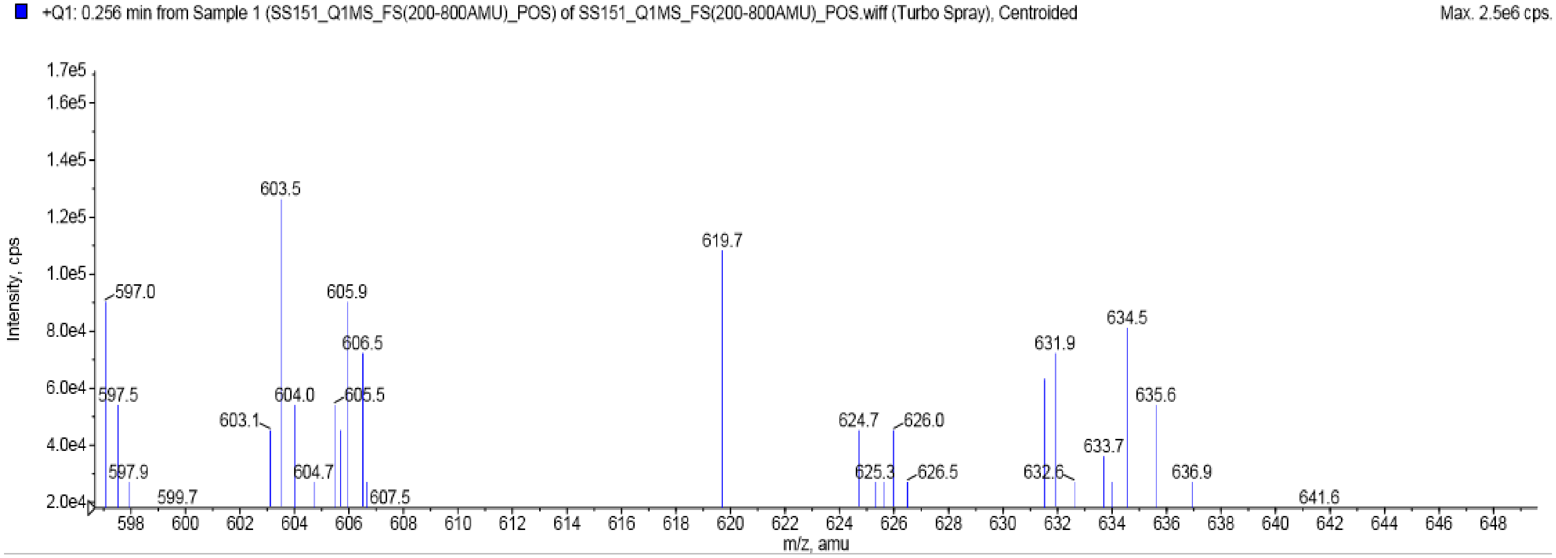

Figure S9 Mass spectrum of [Zn(TMPIMP)2].2H_2O

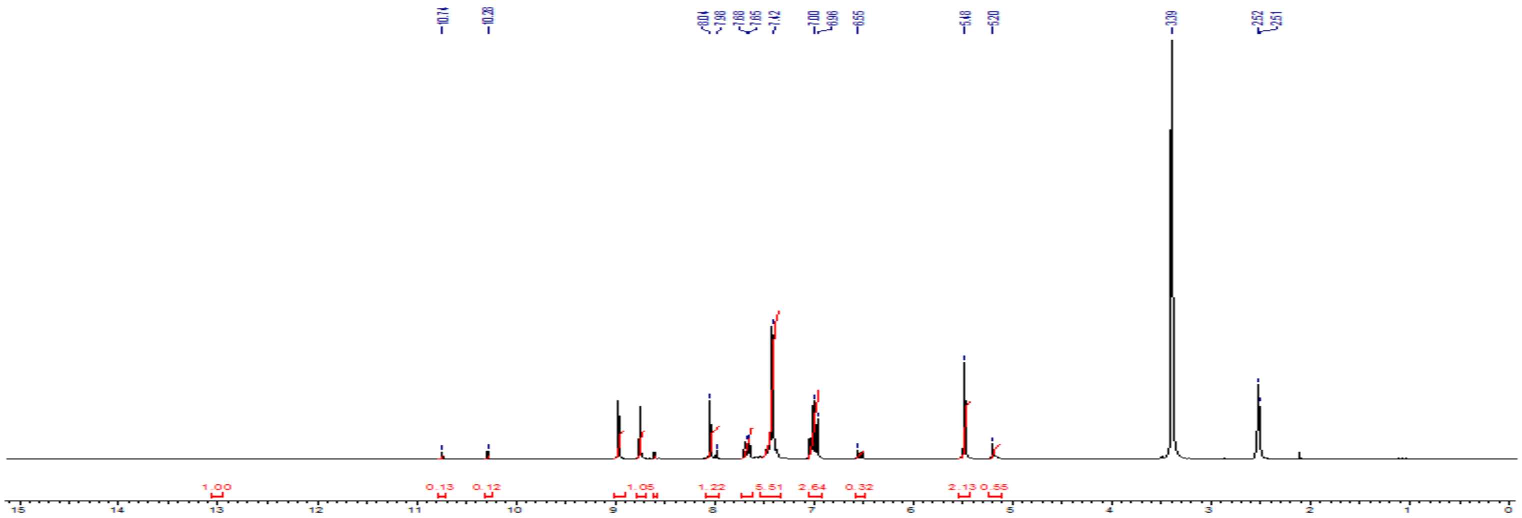

Figure S10 ^{1}H NMR spectrum of $[Zn(TMPIMP)_2].2H_2O$

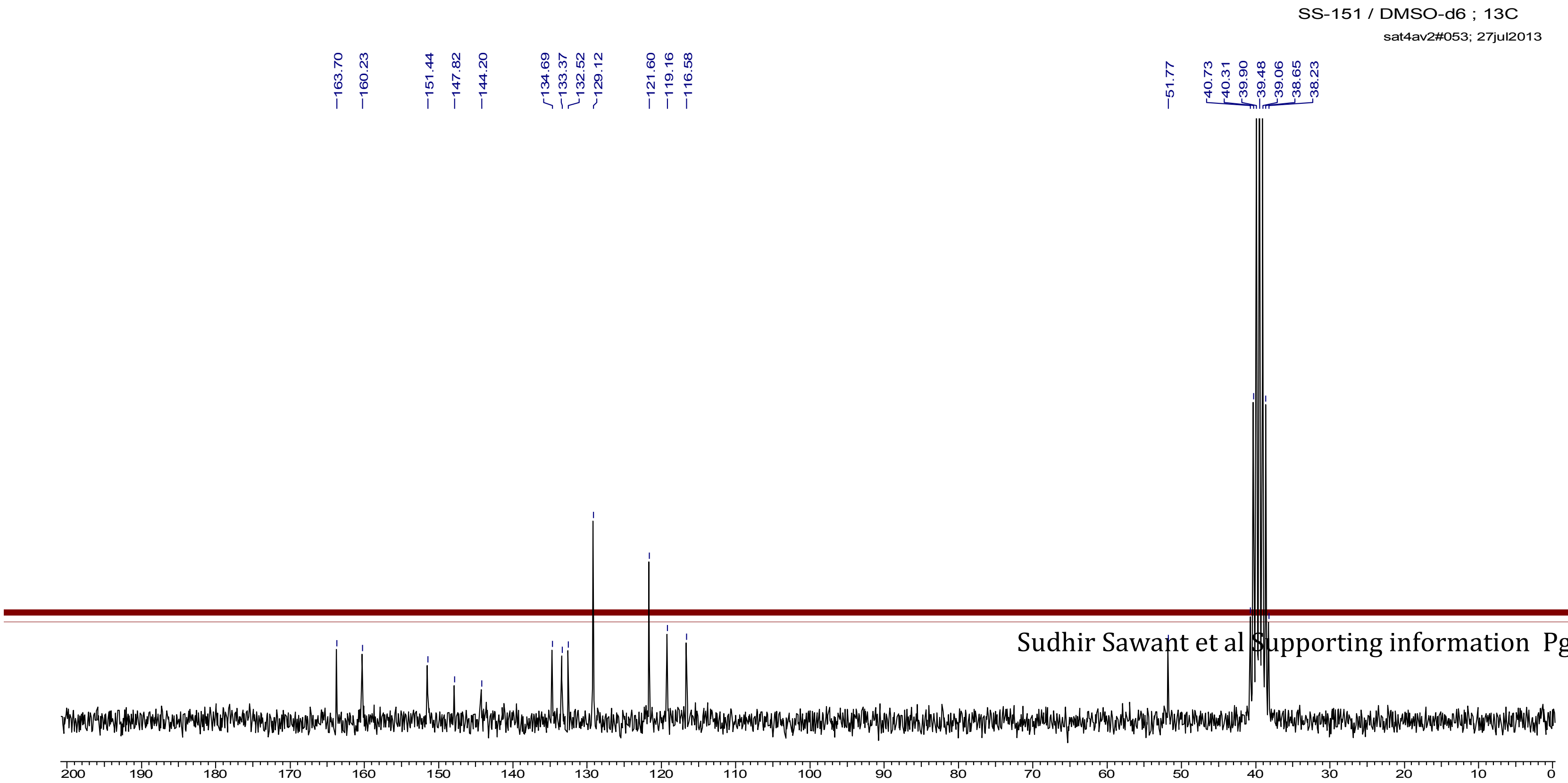

Figure S11 ^{13}C NMR spectrum of $[Zn(TMPIMP)_2].2H_2O$

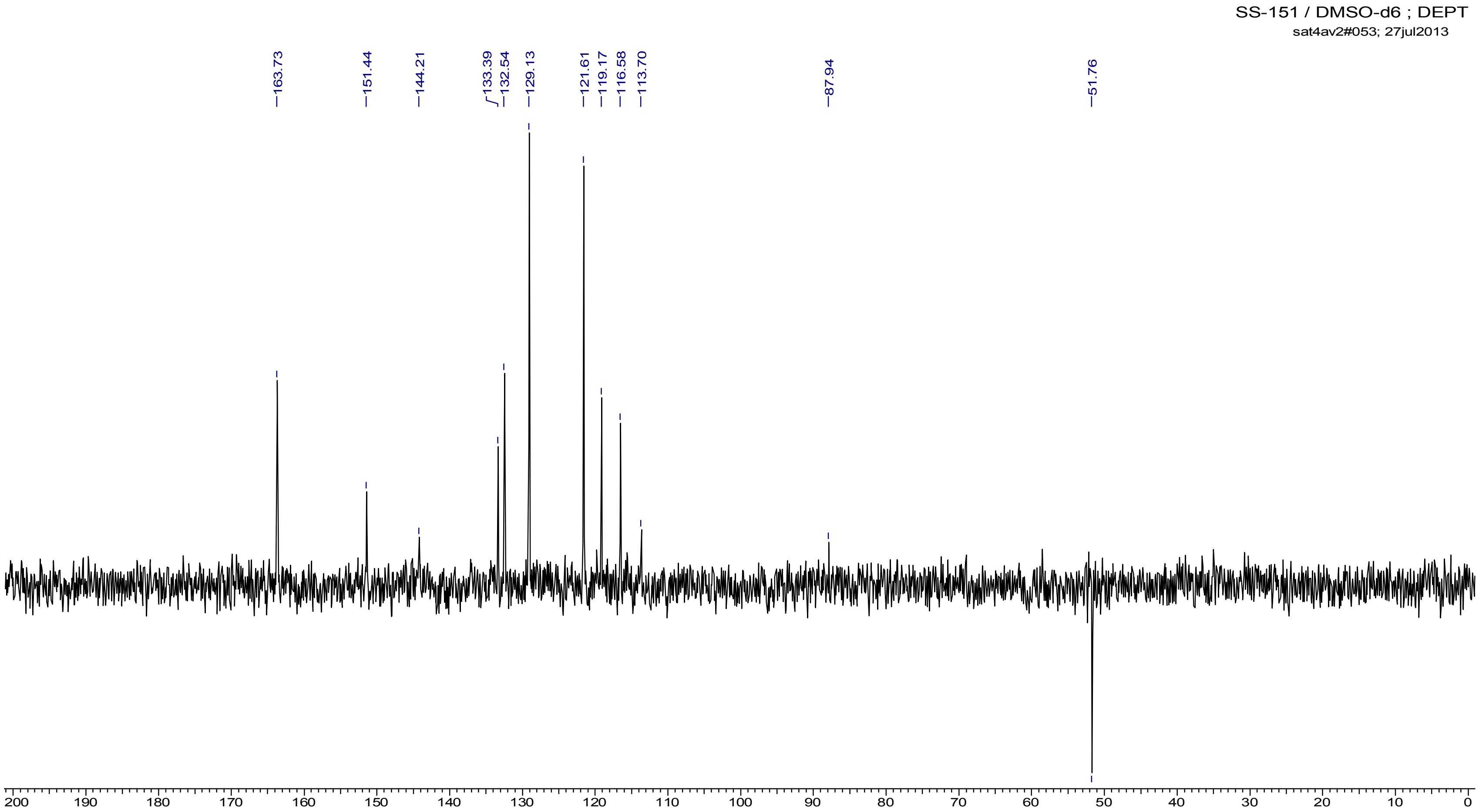

Figure S12 ^{13}C NMR DEPT spectrum of $[Zn(TMPIMP)_2].2H_2O$

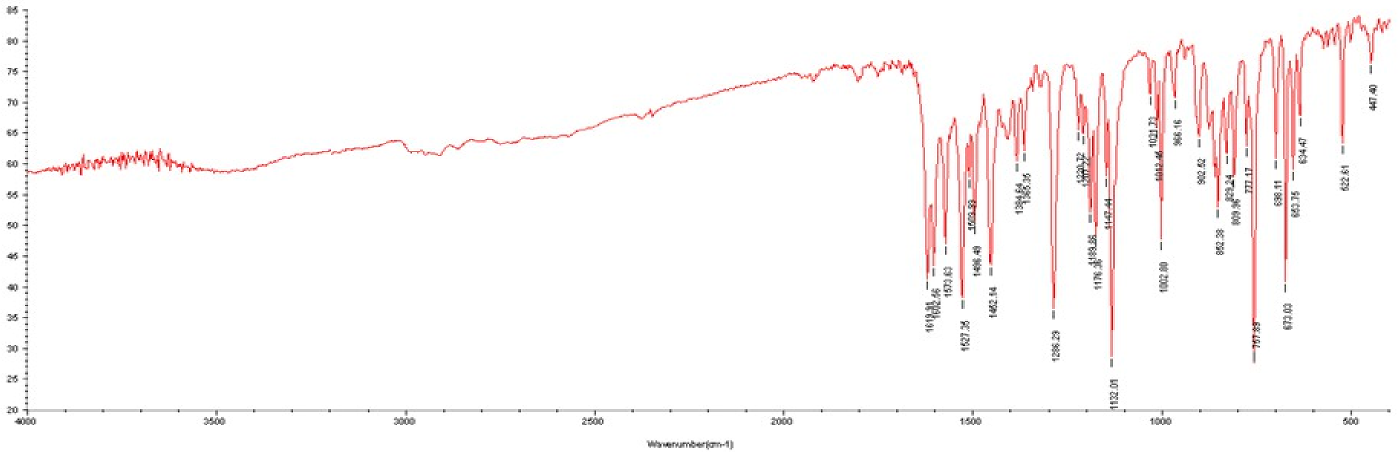

Figure S13 IR spectrum of [Zn(TMPIMP)$_2$].2H_2O

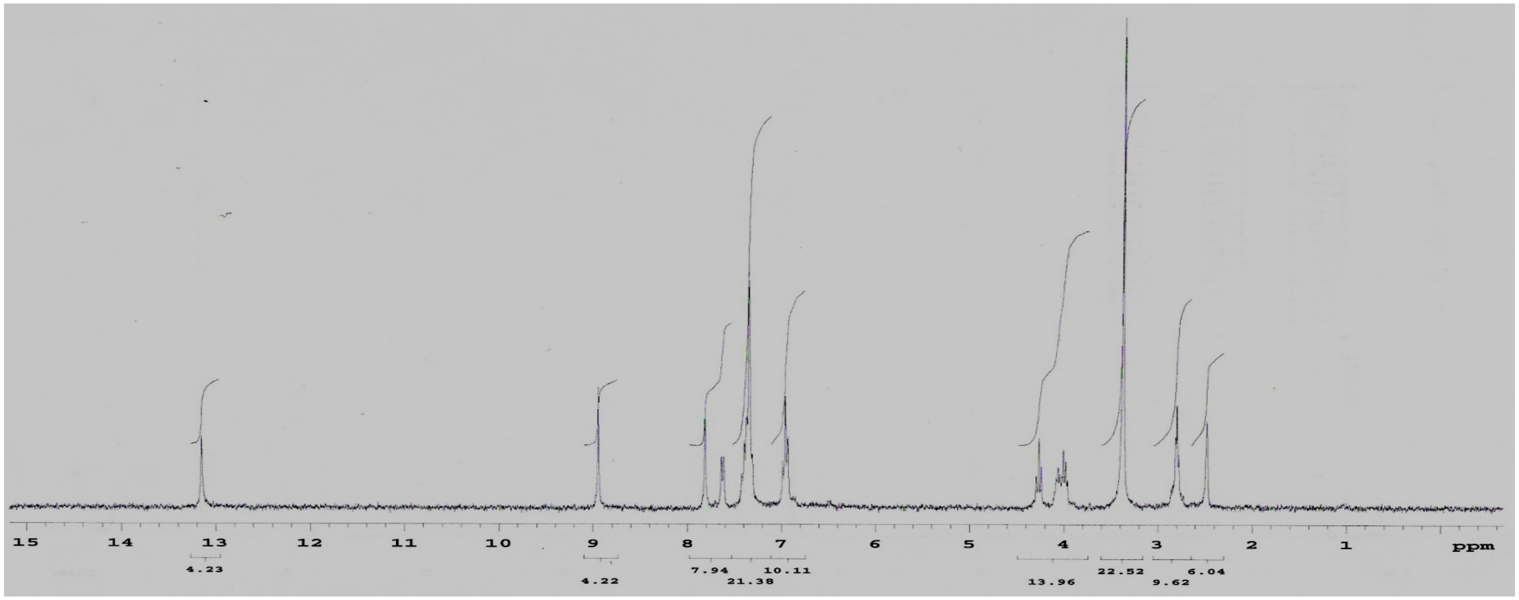

Figure S14 ^{1}H NMR Spectrum of Schiff base HBABO

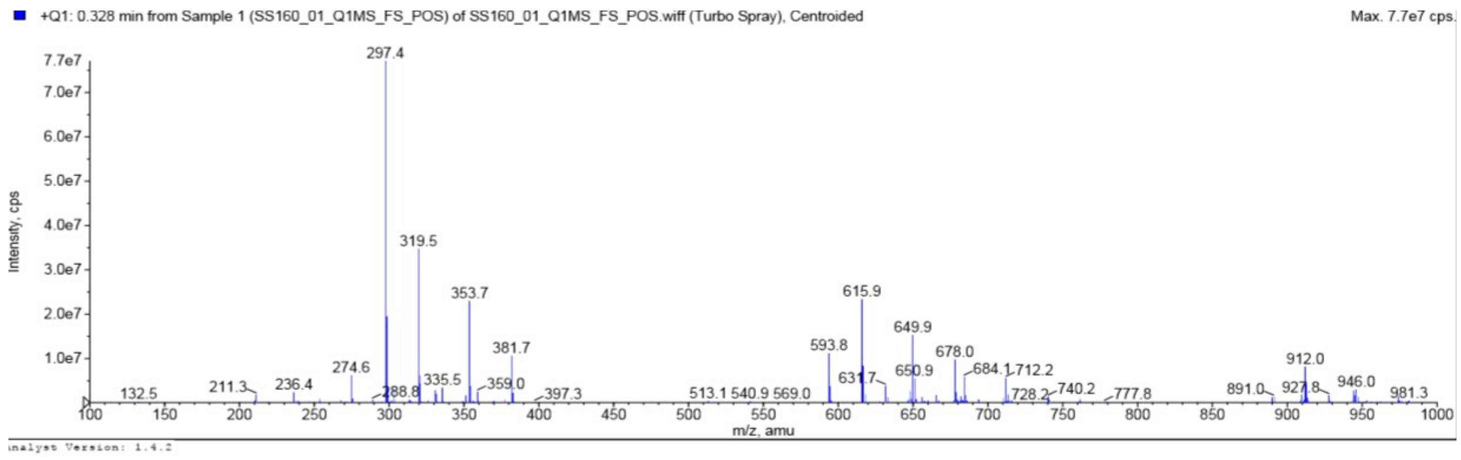

Figure S15 MS Spectrum of HBABO Schiff base

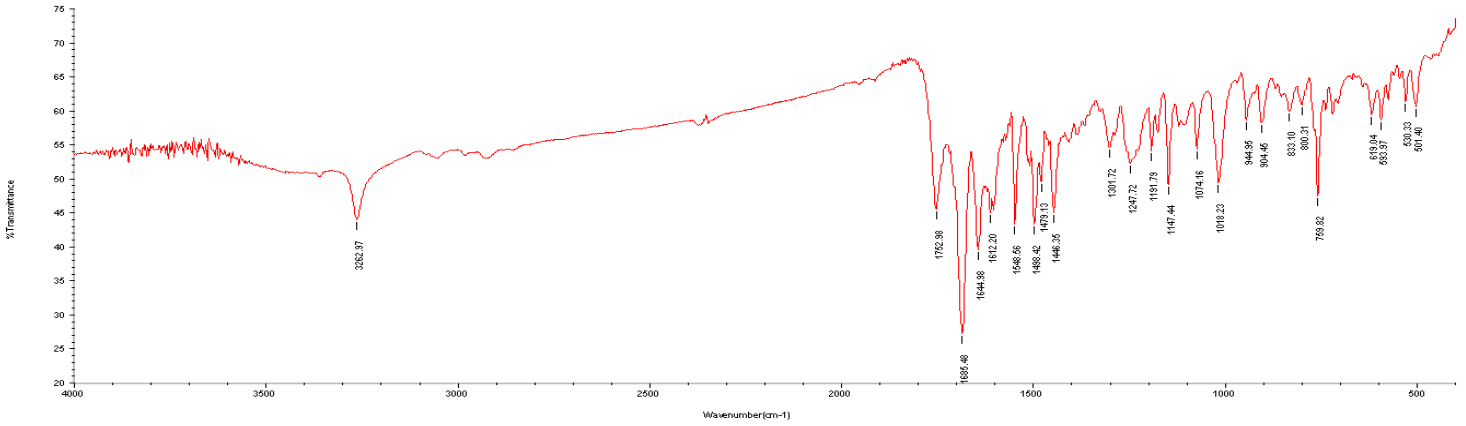

Figure S16 IR Spectrum of HBABO Schiff base

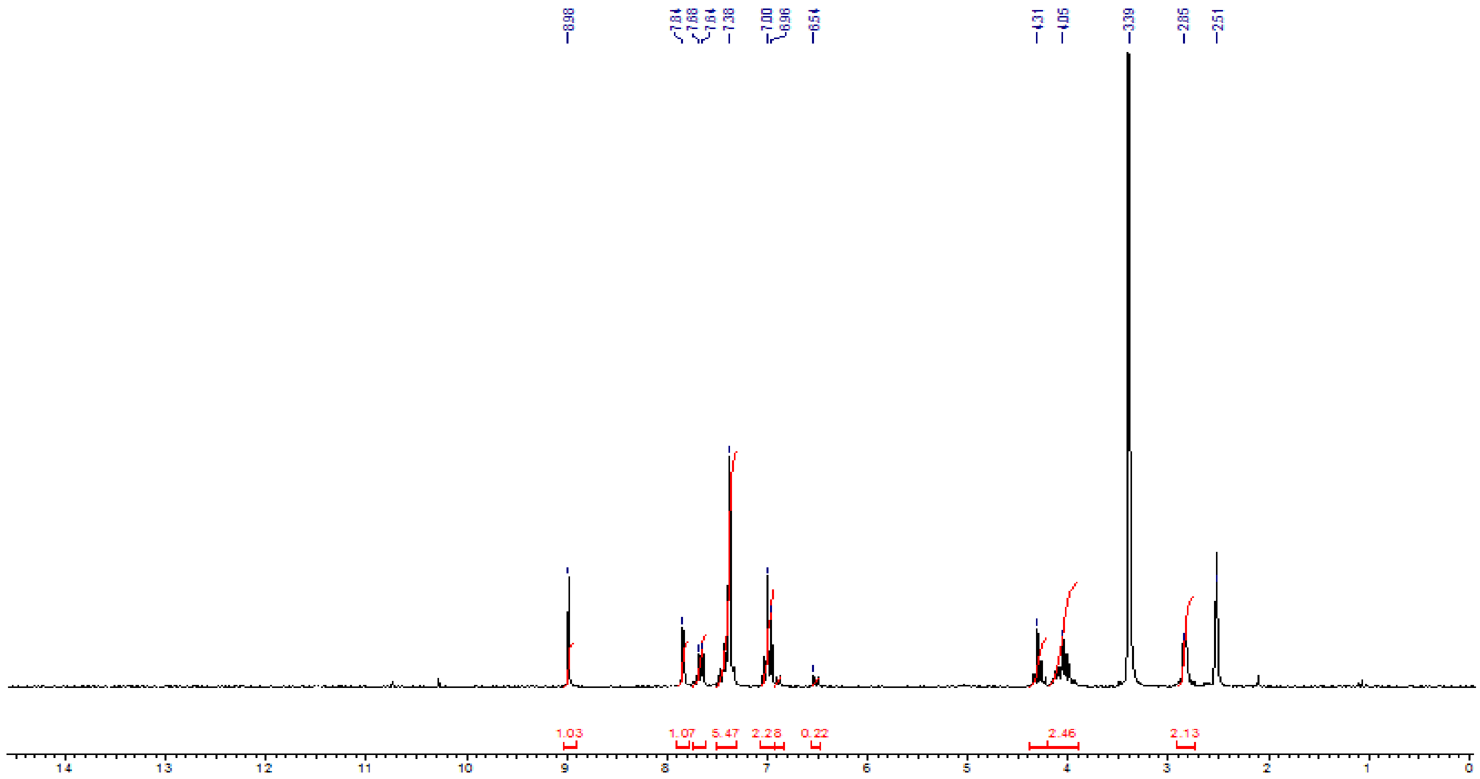

Figure S17 ^{1}H NMR Spectrum of Zn(HBABO)$_2$.2H_2O

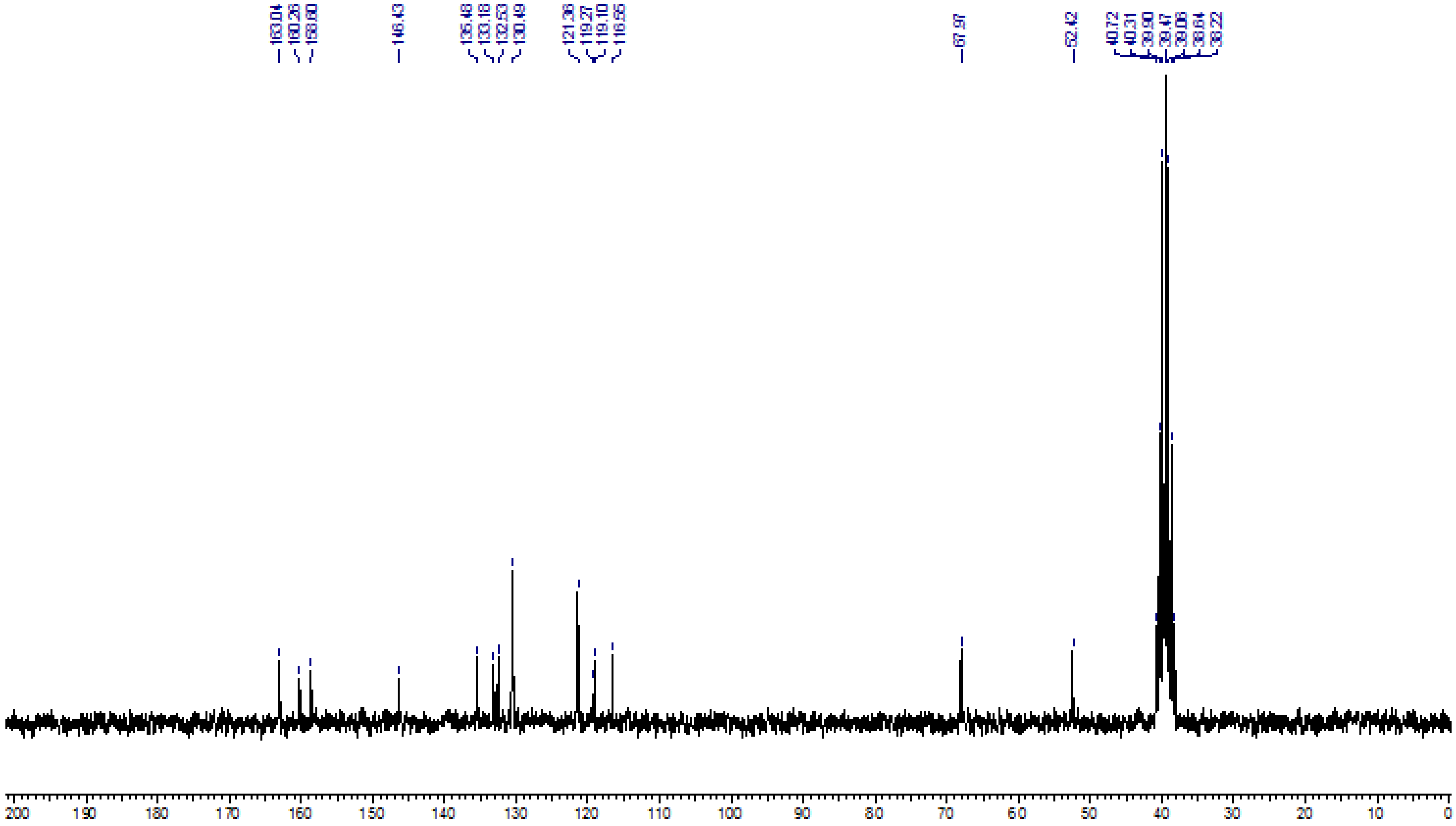

Figure S18 ^{13}C NMR Spectrum of $Zn(HBABO)_2.2H_2O$

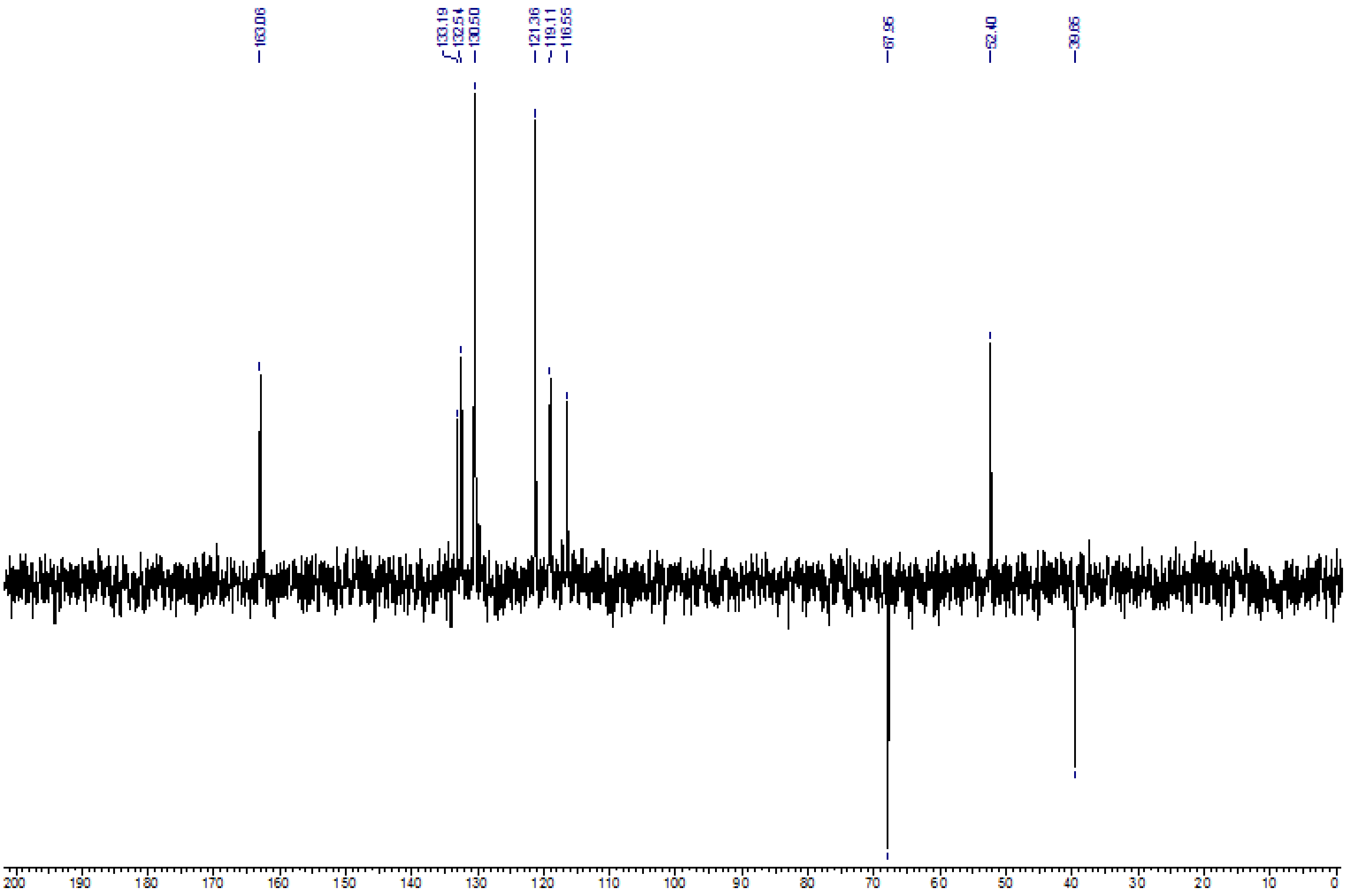

Figure S19 ^{13}C NMR DEPT Spectrum of $Zn(HBABO)_2.2H_2O$

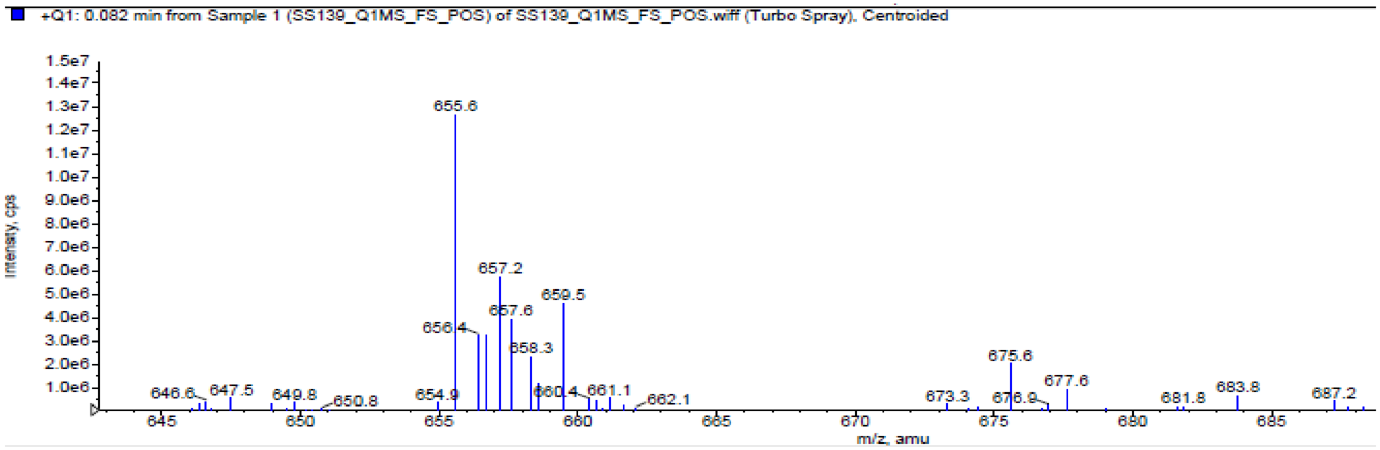

Figure S20 MS Spectrum of $Zn(HBABO)_2.2H_2O$

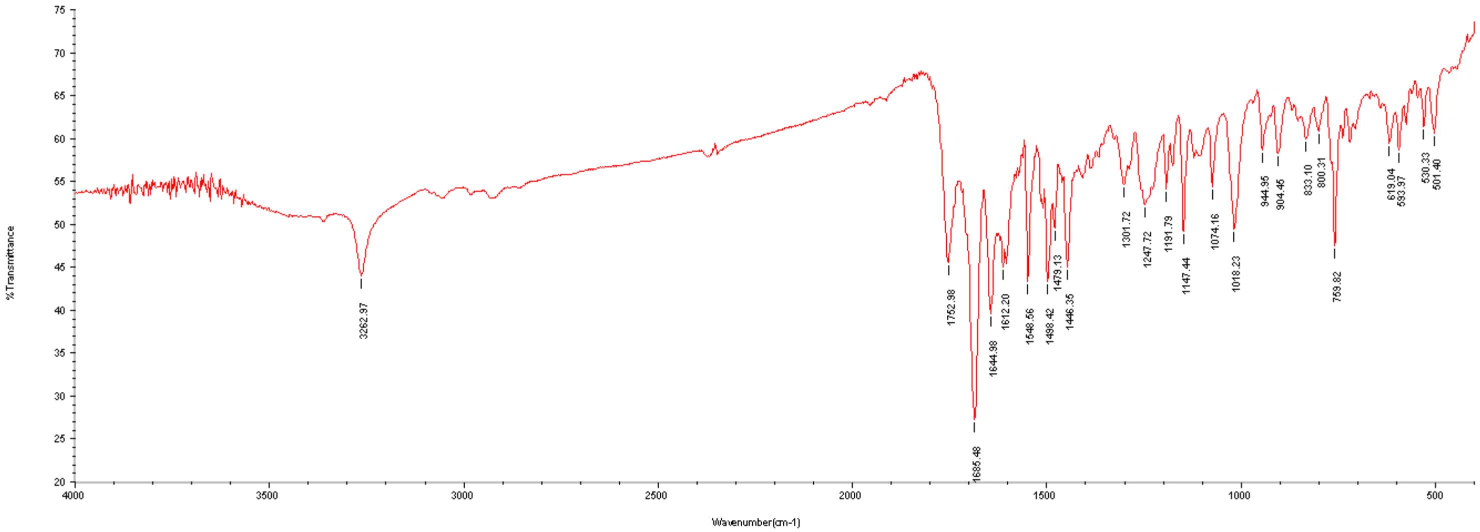

Figure S21 IR Spectrum of $Zn(HBABO)_2.2H_2O$

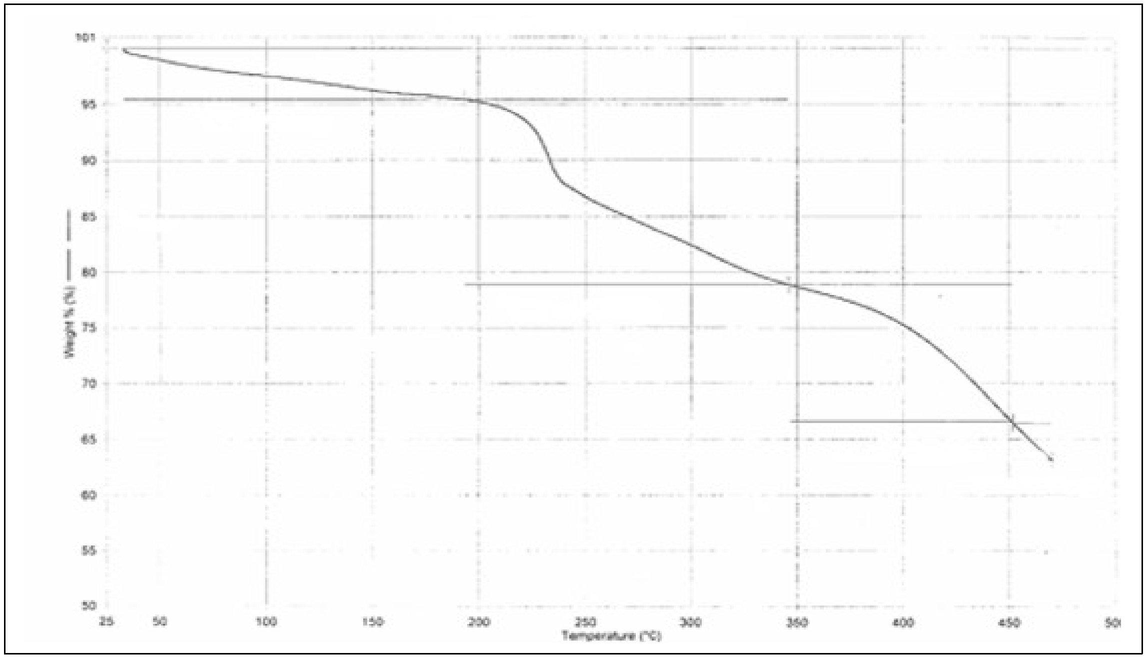

Figure S22 TGA graph of Zn(NMAPIMHMC)$_2$.2H_2O

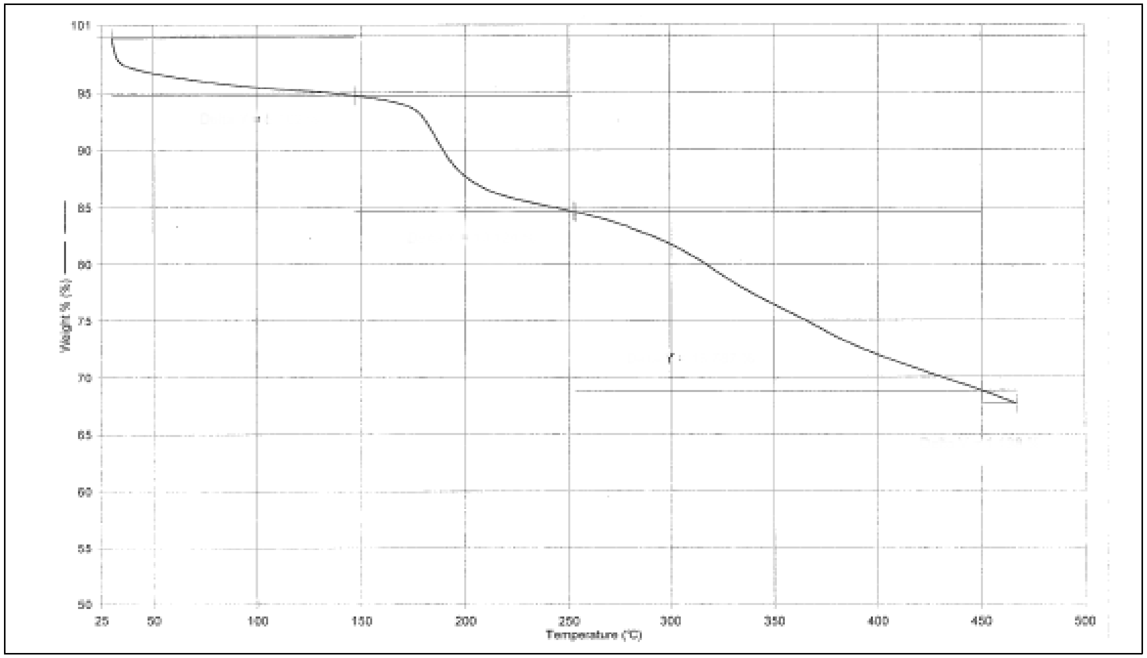

Figure S23 TGA graph of $Zn(TMPIMP)_2.2H_2O$

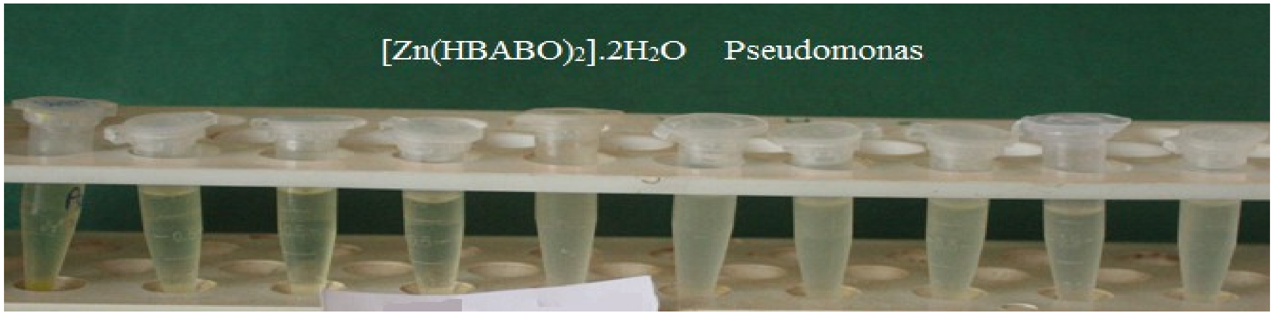

Figure S24 Antibacterial activity of $[Zn(HBABO)_2].2H_2O$ against *Pseudomonas*

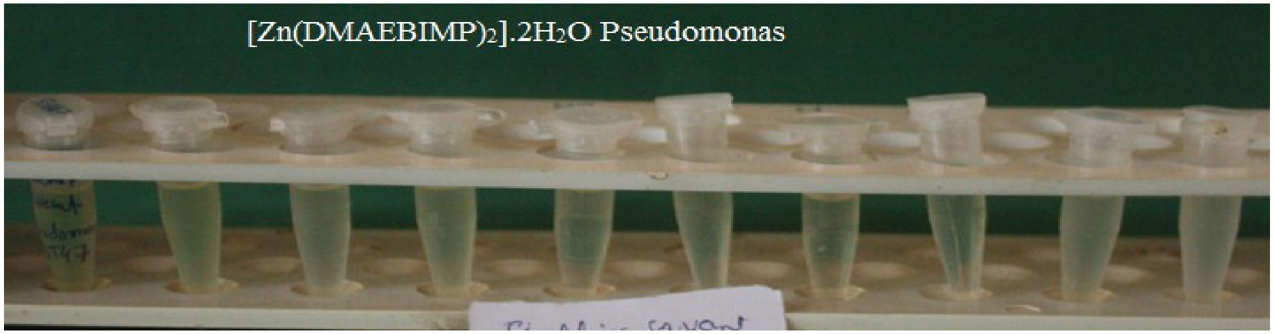

Figure S25 Antibacterial activity of $[Zn(DMAEBIMP)_2].2H_2O$ against *Pseudomonas*

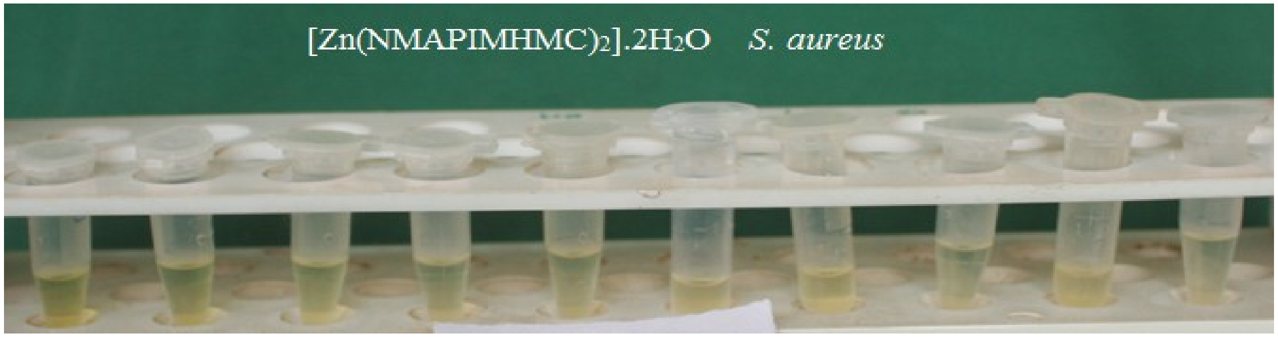

Figure S26 Antibacterial activity of $[Zn(NMAPIMHMC)_2].2H_2O$ against *S. aureus*

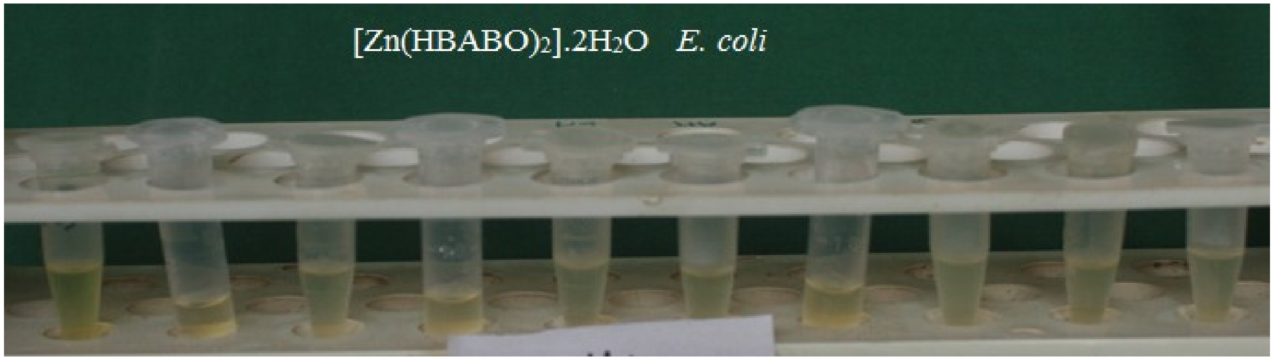

Figure S27 Antibacterial activity of $[Zn(HBABO)_2].2H_2O$ against *E.Coli*

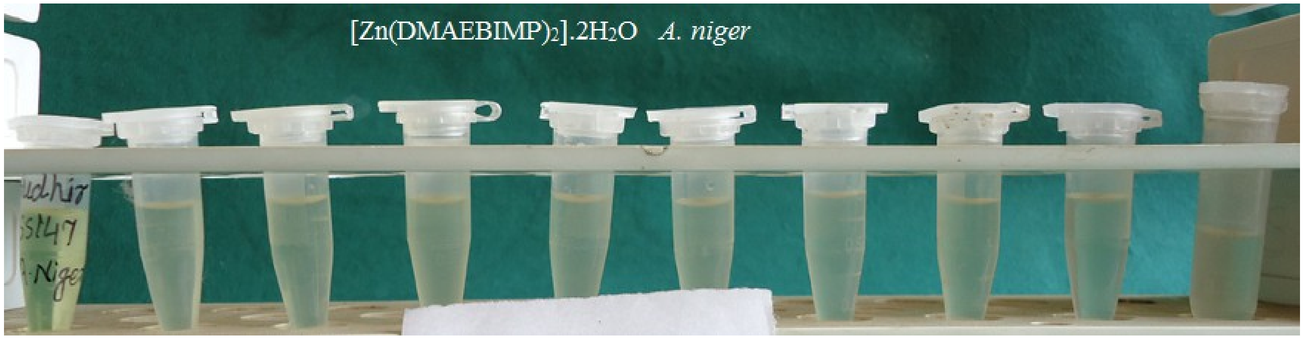

Figure S28 Antifungal activity of [Zn(DMAEBIMP)$_2$].2H_2O against *A.niger*

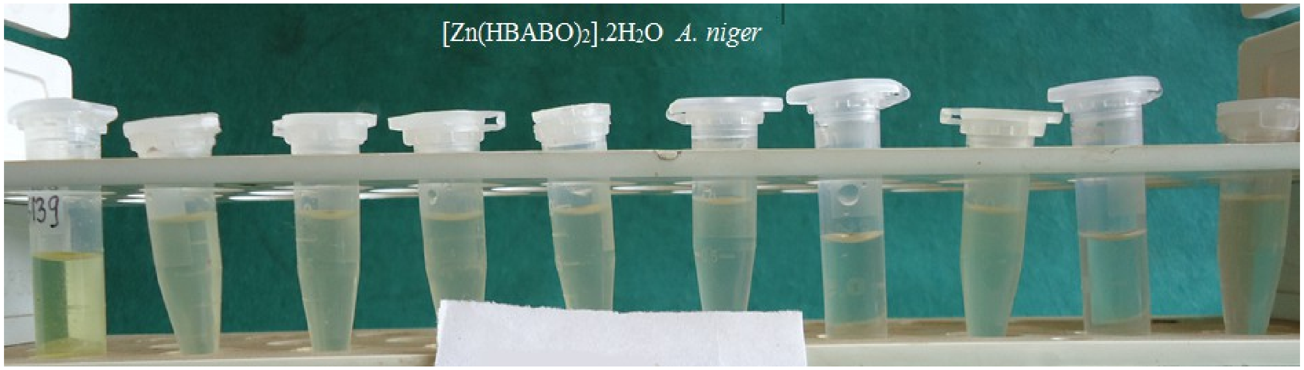

Figure S29 Antifungal activity of [Zn(HBABO)$_2$].2H_2O against *A.niger*

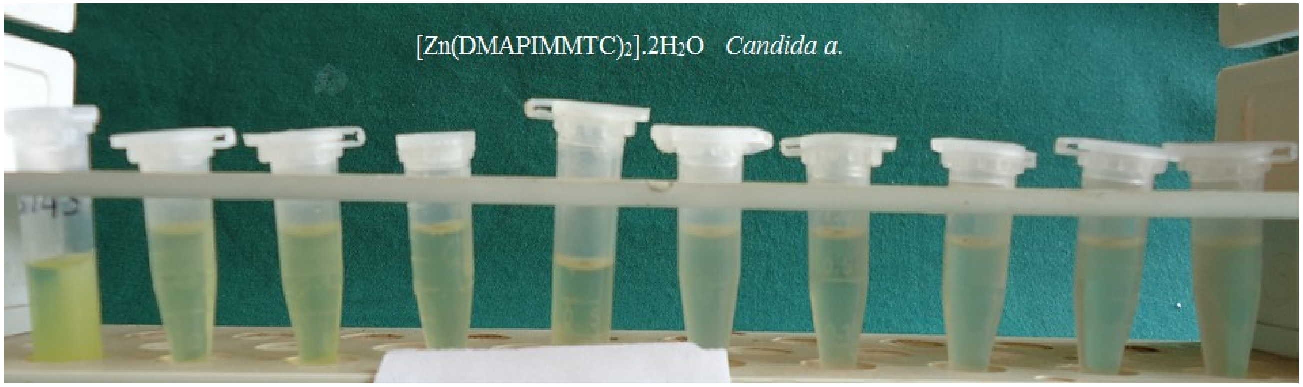

Figure S30 Antifungal activity of [Zn(DMAPIMMTC)$_2$].2H_2O against *Candida a.*
